# Overcoming inefficiencies arising due to the impact of the modifiable areal unit problem on single-aggregation disease maps

**DOI:** 10.1186/s12942-020-00236-y

**Published:** 2020-10-03

**Authors:** Matthew Tuson, Matthew Yap, Mei Ruu Kok, Bryan Boruff, Kevin Murray, Alistair Vickery, Berwin A. Turlach, David Whyatt

**Affiliations:** 1 Medical School, University of Western Australia, Perth, Australia; 2 Department of Mathematics and Statistics, University of Western Australia, Perth, Australia; 3 UWA School of Agriculture and Environment, University of Western Australia, Perth, Australia; 4 Department of Geography, University of Western Australia, Perth, Australia; 5 School of Population and Global Health, University of Western Australia, Perth, Australia

**Keywords:** Disease mapping, Modifiable areal unit problem, Single-aggregation disease maps, Zonation-dependence, Resource allocation efficiency

## Abstract

**Background:**

In disease mapping, fine-resolution spatial health data are routinely aggregated for various reasons, for example to protect privacy. Usually, such aggregation occurs only once, resulting in ‘single-aggregation disease maps’ whose representation of the underlying data depends on the chosen set of aggregation units. This dependence is described by the modifiable areal unit problem (MAUP). Despite an extensive literature, in practice, the MAUP is rarely acknowledged, including in disease mapping. Further, despite single-aggregation disease maps being widely relied upon to guide distribution of healthcare resources, potential inefficiencies arising due to the impact of the MAUP on such maps have not previously been investigated.

**Results:**

We introduce the overlay aggregation method (OAM) for disease mapping. This method avoids dependence on any single set of aggregate-level mapping units through incorporating information from many different sets. We characterise OAM as a novel smoothing technique and show how its use results in potentially dramatic improvements in resource allocation efficiency over single-aggregation maps. We demonstrate these findings in a simulation context and through applying OAM to a real-world dataset: ischaemic stroke hospital admissions in Perth, Western Australia, in 2016.

**Conclusions:**

The ongoing, widespread lack of acknowledgement of the MAUP in disease mapping suggests that unawareness of its impact is extensive or that impact is underestimated. Routine implementation of OAM can help avoid resource allocation inefficiencies associated with this phenomenon. Our findings have immediate worldwide implications wherever single-aggregation disease maps are used to guide health policy planning and service delivery.

## Background

The practice of disease mapping is fundamental to public health [1], with disease maps currently being produced by healthcare organisations worldwide, including the World Health Organisation [2].

A fundamental function of a disease map is to guide geographically-prioritised resource allocation. As such, disease maps have been used to guide the spatial targeting of interventions to address HIV, cholera, Ebola, and malaria [3–11], for example. Disease maps have also been used to guide geographically-targeted responses to the current global COVID-19 pandemic. In Italy, for example, mass COVID-19 testing in the town of Vo proved effective in identifying asymptomatic carriers of the virus, thereby limiting its further spread [12]. Similarly, in Australia, geographically-localised testing has been carried out in areas with elevated risk of community transmission [13–15].

To evaluate a disease map’s utility for this purpose, consideration of its expected ‘efficiency’ is critical. In this context, efficiency has two key aspects: ‘targeting efficiency’ and ‘logistical efficiency’. The targeting efficiency of a given map can be defined by the percentage of cases that could hypothetically be reached, or ‘intervened with’ through targeting a certain percentage of the study denominator (usually population). For example, a recent study describing the planned geographically-prioritised distribution of a limited supply of oral cholera vaccines in sub-Saharan Africa [8] proposed that regions with high rates of cholera be prioritised for distribution. On the other hand, the ‘logistical efficiency’ of a disease map can be represented by the number of distinct geographic ‘target regions’. Since both intervention resources and the capacity of authorities to intervene are generally limited, it is essential to balance the targeting and logistical efficiency of a given disease map.

In practice, disease maps are often produced at a single, more-coarse resolution than that at which data were collected or made available (the ‘minimal’ resolution). This is in order to overcome issues associated with examining fine-resolution data, including: (i) violation of privacy, (ii) infeasible computation, and (iii) the small number problem, which manifests in unstable estimates of disease risk [16]. Since data underlying such maps are essentially an aggregation of minimal-resolution data, they can be termed ‘single-aggregation disease maps’. In Australia, for example, population data are obtainable by Statistical Areas Level 1 (SA1; mean population size approximately 400 residents [17]), but disease maps are usually produced by larger areas such as SA2 (between approximately 3,000 and 25,000 residents) or SA3 (between approximately 30,000 and 130,000 residents) [18–20]. This is partly due to extensive ethical clearances limiting access to fine-resolution health data, and partly due to the ‘small-number’ issues listed above. Similarly, in the US and UK, for example, disease maps are often produced by either county or census ward, despite finer-resolution data being obtainable [21, 22]. As a third example, the previously-cited study of cholera used a grid of 20 × 20 km units in preference to potentially finer-resolution grids, in part due to computational constraints [8].

Unfortunately, single-aggregation disease maps are undermined through being dependent on the chosen set of aggregate-level mapping units. This dependence is described by the modifiable areal unit problem (MAUP). First described in the literature in 1934 [23], and coined in 1979 [24], the MAUP has since been widely investigated in geography, statistics, and elsewhere. In disease mapping, the MAUP describes how mapped values depend on both the scale of spatial aggregation (the scale aspect) and the position of boundaries between spatial units (the zonation aspect) [25]. These aspects manifest in various misleading phenomena; for example, disease clusters that are relatively small compared to the chosen units, or located where several units intersect, might not be detected.

Despite its extensive literature, in practice, the MAUP is rarely acknowledged, including in disease mapping. This is evidenced by a 2014 review, which noted that the MAUP was recognised in only 1% of papers using spatially aggregated data [26]. More recently, there is little evidence to suggest acknowledgement of the MAUP has increased, with papers containing single-aggregation disease maps but not acknowledging the MAUP constantly being published in top-ranked journals (e.g. see [27–29]). On the other hand, papers that do acknowledge the MAUP often simply demonstrate how different results can be obtained through examining administrative units at different scales (e.g. see [30]). This is consistent with a general tendency of the literature to examine the MAUP’s scale aspect, as opposed to its zonation aspect [31]. While some association studies *have* examined the impact of the MAUP’s zonation aspect (e.g. see [24, 32, 33]), to our knowledge, no similar study exists in disease mapping. Further, as far as we are aware, no previous study has investigated the impact of the MAUP in the context of efficiency. Such an investigation is critically required, given ubiquitous reliance on single-aggregation disease maps to guide distribution of limited healthcare resources.

Therefore, we introduce the overlay aggregation method (OAM) for disease mapping. Extending a recently suggested approach [33], OAM combines many aggregate-level disease maps to derive a single, minimal-resolution map. Using simulated data, we characterise OAM as both: i) a novel framework to systematically quantify the impact of the MAUP’s zonation aspect on single-aggregation disease maps, and ii) a novel smoothing technique that overcomes both this impact and the small-number issues listed previously. We compare OAM to single-aggregation disease maps by their targeting and logistical efficiency, and demonstrate how OAM effectively balances those aspects. We then apply OAM to a real-world dataset: ischaemic stroke hospital admissions (stroke) in Perth, Western Australia (WA), in 2016. Strokes require rapid intervention to avoid irreparable nerve tissue damage [34], necessitating ongoing consideration of patients’ access to essential stroke services such as specialist hospital units or ambulance depots. Understanding the spatial distribution of stroke supports this endeavour through guiding placement of such services.

## Methods

### Overlay Aggregation Method (OAM)

OAM comprises five steps:

1. Define the minimal units;
2. Set parameter values:
  - The number of aggregate-level maps; and
  - Their geographical scale of aggregation;
3. Create multiple sets of aggregate-level mapping units, or ‘zonations’;
4. Create aggregate-level disease maps based on those zonations; and
5. Combine the aggregate-level disease maps to produce a single, minimal-resolution map.

These steps are described in detail below.

#### Step 1. Define the minimal units

Since its first step involves specifying a set of minimal units, OAM operates in the same way as applied to either truly areal data (e.g. such as that often collected in surveys) or data spatially aggregated from an original point location form. This specification will often be guided by data availability; in Australia, for example, SA1s might often be defined as the minimal units due to health data generally not being obtainable below that resolution. By contrast, if point location data were available, then minimal units substantially smaller than SA1 (or their equivalent in another country) could be defined.

#### Step 2. Set parameter values

The number of maps and their geographical scale of aggregation together govern the degree of smoothing in OAM’s output. We will show how one might choose an appropriate number of maps guided by observation of a minimal degree of change in OAM’s output as the number of maps is increased. By contrast, the choice of scale should be guided by the characteristics of a planned intervention (see Sect. Discussion).

#### Step 3. Create the aggregate-level zonations

OAM’s zonations must completely segment the geographical study area. Following previous authors [32, 33], we show how suitable zonations can be created using the freely-available software AZTool [35, 36]. Briefly, AZTool operates by zoning a set of minimal units according to a target denominator size and potentially other constraints (e.g. minimum and maximum threshold denominator sizes). To create OAM’s zonations, only specification of target and minimum threshold denominator sizes is required.

#### Step 4. Create the aggregate-level disease maps

Any model deemed appropriate by the user can be fitted to OAM’s zonations; OAM in no way advises the choice of model, since its focus is the appropriate *preparation* of available data, rather than its modelling. For the simulated dataset, we created crude rate maps for each of OAM’s zonations, while for stroke, we fitted the widely implemented Besag, York & Mollie (BYM) model [37, 38]. The latter modelling was undertaken using the Integrated Nested Laplace Approximation (INLA) approach [39], implemented in the R [40] package INLA (https://www.r-inla.org). INLA allows for approximate Bayesian inference in latent Gaussian models. For model formulae and R code, see [41].

#### Step 5. Combine the aggregate-level disease maps

In this step, minimal unit values are derived through weighting the values of aggregate-level units in which they are comprised (one per zonation), using the formula:

1 $${v}_{m}=\frac{\sum_{p \in P}\left[\frac{{d}_{m}}{{d}_{p}}\times {v}_{p}\right]}{\sum_{p \in P}\left[\frac{{d}_{m}}{{d}_{p}}\right]}$$

where $p$ indexes the set $P$ of aggregate-level units comprising minimal unit $m$; ${d}_{m}$ and ${d}_{p}$ are the denominator values for units $m$ and $p$, respectively; ${v}_{p}$ is the value of unit $p$; and ${v}_{m}$ is the denominator-weighted mean value derived for unit $m$. For both the simulation and stroke, ${d}_{m}$ and ${d}_{p}$ are population sizes. For the simulation, ${v}_{p}$ is the crude rate of unit $p$ and ${v}_{m}$ is the population-weighted mean crude rate of unit $m$. For stroke, ${v}_{p}$ is the estimated relative risk (RR) of unit $p$ and ${v}_{m}$ is the population-weighted mean RR of unit $m$.

Equation (1) is undefined for minimal units with zero denominator values. Therefore, to maintain the smooth appearance of the final map, ${d}_{m}$ can be cancelled in Eq. (1) to give:

2 $${v}_{m}=\frac{\sum_{p \in P}\left[\frac{1}{{d}_{p}}\times {v}_{p}\right]}{\sum_{p \in P}\left[\frac{1}{{d}_{p}}\right]}$$

where all parameters are as defined previously. Equation (2) will output positive values for minimal units with zero denominators and identical values to Eq. (1) otherwise. All OAM results presented below were derived using Eq. (2).

### Simulation

A point location dataset of 100 disease cases was generated through specifying a spatially-correlated random field and assigning cases across that field according to a multinomial probability distribution. We suppose that population size is the denominator of interest, with population data available at the resolution of a 100 × 100 unit grid where each cell has a population size of one. Thus, the total population size is 10,000. We define a 20 × 20 unit grid to be the set of minimal units; thus, each minimal unit has a population size of 25. Note: population size is defined to be the denominator due to this being the most common situation in practice; however, other denominators, e.g. geographical area, could be used.

We produced three maps of the simulated dataset:

i) A minimal-resolution map of crude rates;
ii) A single-aggregation map of crude rates, based on a 5 × 5 unit grid (population size: 400 per unit); and
iii) A minimal-resolution map of population-weighted mean crude rates, using OAM.

We used AZTool to create 100 aggregate-level zonations for OAM, based on target and minimum threshold population sizes of 400 and 300, respectively. The former value was chosen in order that the single-aggregation and OAM maps be comparable.

### Stroke

#### Study area

2016 Australian Census SA boundaries for Perth were obtained from the Australian Bureau of Statistics (ABS). Perth was defined to comprise the four Greater Perth SA4s: ‘Perth—Inner’, ‘Perth—South East’, Perth—South West’, ‘Perth—North East’, and ‘Perth—North West’, excluding two single-SA1 islands: Rottnest Island and Garden Island. The latter were excluded due to Rottnest Island operating primarily as a tourist and day-trip destination and Garden Island housing the Australian Navy’s largest fleet base. Sets of minimal and single-aggregation units were defined to be SA1 and SA2, respectively.

#### Outcome data

Stroke records for Perth in 2016 were extracted from the WA Hospital Morbidity Data Collection (HMDC) [42] and aggregated by SA1. Following [43], we defined stroke admissions to be those with a principal International Statistical Classification of Diseases and Related Health Problems, tenth revision, Australian Modification (ICD-10-AM) [44] diagnosis of I63 (Cerebral infarction), I64 (Stroke, not specified as haemorrhage or infarction), or H34.1 (Central retinal artery occlusion). In total, 3,534 stroke admissions were extracted for WA, of which 11 (0.3%) were excluded due to not having an SA1 of residential address recorded. A further 996 were excluded due to occurring among individuals residing outside of Perth (i.e. in regional WA), and of the remaining admissions, four (0.2%) were excluded due to occurring in SA1s with zero population sizes as reported by the ABS. Thus, a total of 2,523 admissions were available for analysis.

#### Population data

Population data for the 2016 Australian Census were extracted from the ABS, stratified by SA1.

#### Analysis

Mirroring the simulation analysis, we produced:

i) An SA1-resolution map of RRs;
ii) An SA2-resolution map of RRs; and
iii) An SA1-resolution map of population-weighted mean RRs, using OAM.

We again used AZTool to create 100 aggregate zonations for OAM, based on target and minimum threshold population sizes of 11,250 and 9,000, respectively. The former value was chosen to approximately match the mean population size of SA2s (11,256). Across all zonations, the median number of SA1s per aggregate unit was 26 (95% quantile interval: 18–33).

### Efficiency

For both the simulation and stroke, we compared maps by their:

i) ‘Targeting efficiency curves’;
ii) ‘Targeting efficiency maps’;
iii) ‘Logistical efficiency curves’; and,
iv) For exemplar ‘target case percentages’, ‘logistical efficiency maps’.

These outputs are described in detail below.

#### Targeting efficiency curves

The targeting efficiency curve for a given disease map plots the cumulative percentage of the denominator ‘targeted’ against the cumulative percentage of cases reached. It is created through ‘targeting’ the mapping units by their values, in descending order.

#### Targeting efficiency maps

A targeting efficiency map is a spatial representation of a targeting efficiency curve. In such maps, units are coloured according to the cumulative percentage of cases they collectively contain, following their target order.

#### Logistical efficiency curves

A logistical efficiency curve plots the number of discontiguous regions requiring targeting to reach different target case percentages (i.e. cumulative percentages of cases). For both the simulation and stroke, discontiguity was determined using rook adjacency.

#### Logistical efficiency maps

A logistical efficiency map displays the target regions associated with a particular target case percentage. It is essentially a dichotomised version of a targeting efficiency map. We created logistical efficiency maps for the simulation and stroke based on exemplar target case percentages of 50% and 15%, respectively.

## Results

### Minimal-resolution analysis of the simulated dataset

**Fig. 1:**
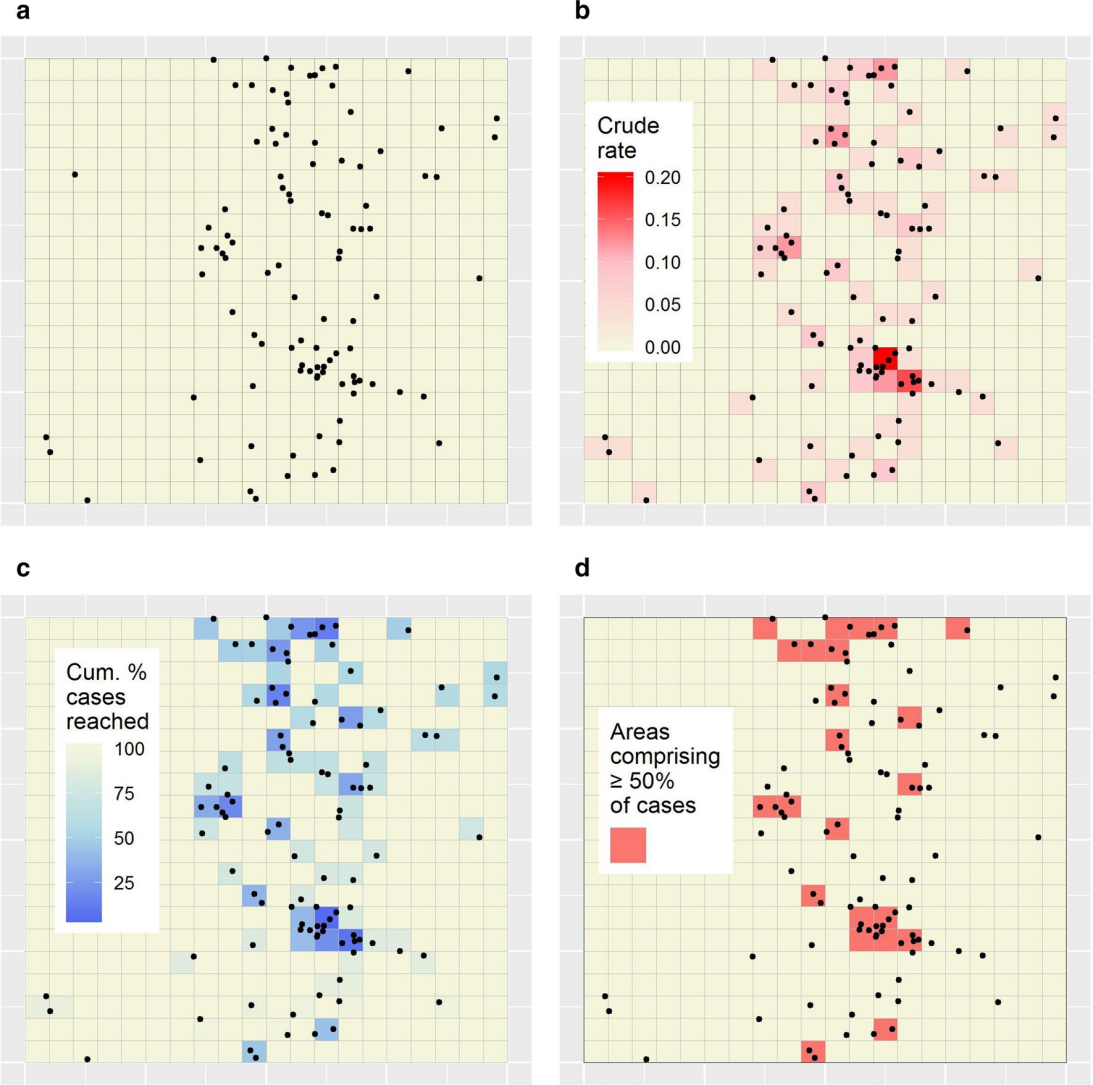
Minimal-resolution efficiency results for the simulated dataset. **a** Simulated point location disease cases with the minimal units overlaid (grey squares).** b** Minimal-resolution disease map of crude rates.** c** Targeting efficiency map associated with **b**.** d** Logistical efficiency map based on a target case percentage of 50%

Figure 1a maps the simulated disease case locations and the minimal units. The cases are broadly grouped in a vertical band to the right of centre of the study area, with variations in grouping within that band.

Figure 1b shows the minimal-resolution disease map of crude rates.

**Fig. 2:**
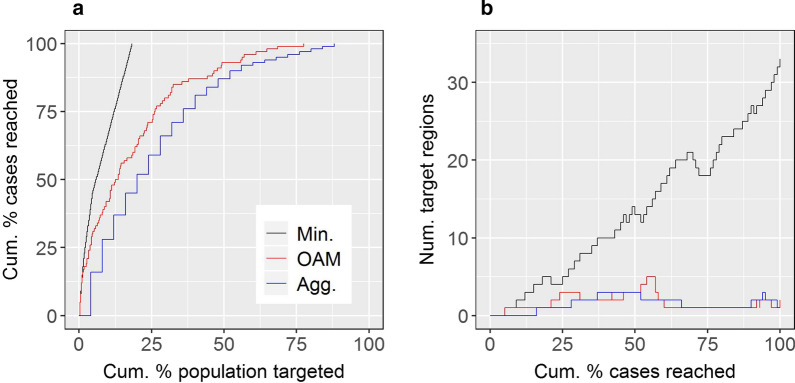
Targeting and logistical efficiency curves for different mapping strategies applied to the simulated dataset. **a** Targeting efficiency curves. **b** Logistical efficiency curves. Curves are shown for the minimal-resolution (Min.); single-aggregation (Agg.); and OAM targeting strategies

Figure 2a shows the targeting efficiency curve associated with Fig. 1b. This curve indicates that 50% of cases could hypothetically be reached through targeting just 23 minimal units, or 5.8% of the population (Table 1).

**Table 1:** Exact efficiency data for the simulation

Mapping method	Cum. % of population targeted	Number of target regions
Min	5.8	13
OAM	12.5	3
Agg	20	2

Shown are cumulative percentage of population targeted and number of target regions values associated with a target case percentage of 50%, for the minimal-resolution (‘Min.’); single-aggregation (‘Agg.’) and OAM targeting strategies.

Figure 1c shows the targeting efficiency map corresponding to Fig. 2a. In this map, the 23 units noted above are coloured blue-to-light blue.

Figure 2b shows the logistical efficiency curve associated with Fig. 1c. This curve indicates that a maximum of 33 regions require targeting to reach any target case percentage (this was for a target case percentage of 100%).

Finally, Fig. 1d shows the logistical efficiency map associated with the target case percentage of 50%. It shows that the 23 units noted above form 13 discontiguous regions (Table 1). Note: the legend indicates that ≥50% of cases are contained within those regions, since, in general, a larger percentage of cases than the specified target might be contained within the regions requiring targeting to reach it. In Fig. 1d the target regions contain exactly 50% of cases, so the ≥ symbol is for illustration only.

### Single-aggregation analysis of the simulated dataset

**Fig. 3:**
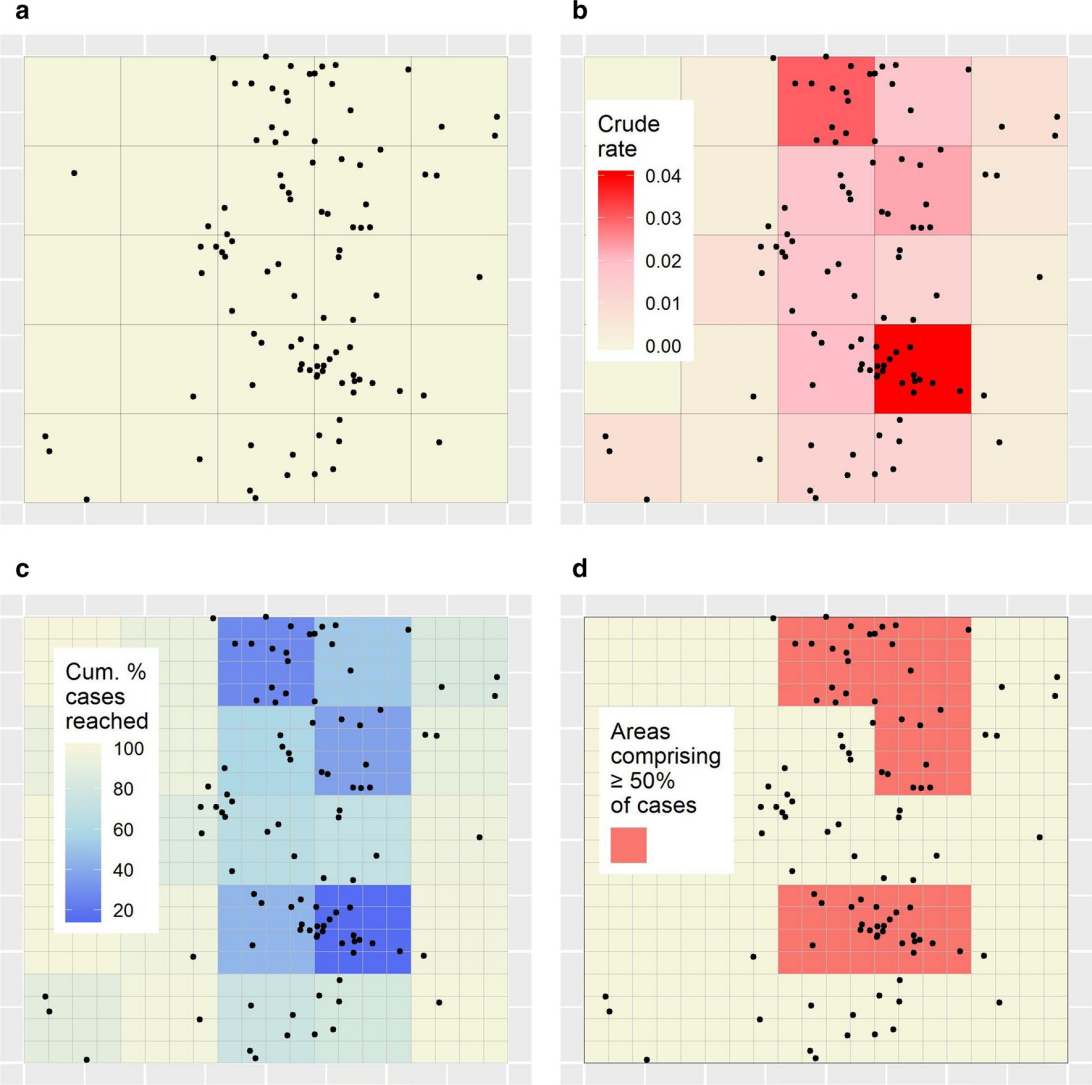
Single-aggregation efficiency results for the simulated dataset. **a** Simulated point location disease cases with the single-aggregation units overlaid (large grey squares). **b** Single-aggregation disease map of crude rates. **c** Targeting efficiency map associated with **b**. **d** Logistical efficiency map based on a target case percentage of 50%

Figure 3a maps the simulated disease case locations and the single-aggregation units. Figure 3b shows the single-aggregation map of crude rates; Fig. 2a the single-aggregation targeting efficiency curve; Fig. 3c the targeting efficiency map; Fig. 2b the logistical efficiency curve; and Fig. 3d the logistical efficiency map based on the target case percentage of 50%. Figure 2 demonstrates decreased targeting efficiency but increased logistical efficiency for the single-aggregation strategy compared to the minimal-resolution result. For example, using the single-aggregation strategy, 50% of cases could hypothetically be reached through targeting 20% of the population within two discontiguous regions (Table 1).

### OAM analysis of the simulated dataset

Clearly, a trade-off exists between the targeting and logistical aspects of efficiency. A minimal-resolution strategy will always maximise targeting efficiency, but at the expense of logistical efficiency. This is the impact of the small number problem. Further, while computational expense is not presently an issue, privacy is likely to be violated by the presentation of rates based on small numbers in Fig. 1b. The single-aggregation strategy overcomes these issues, but at the expense of targeting efficiency. This is the impact of the MAUP. Thus, a targeting strategy is needed that balances the targeting and logistical aspects of efficiency; here, we demonstrate how OAM constitutes such a strategy.

**Fig. 4:**
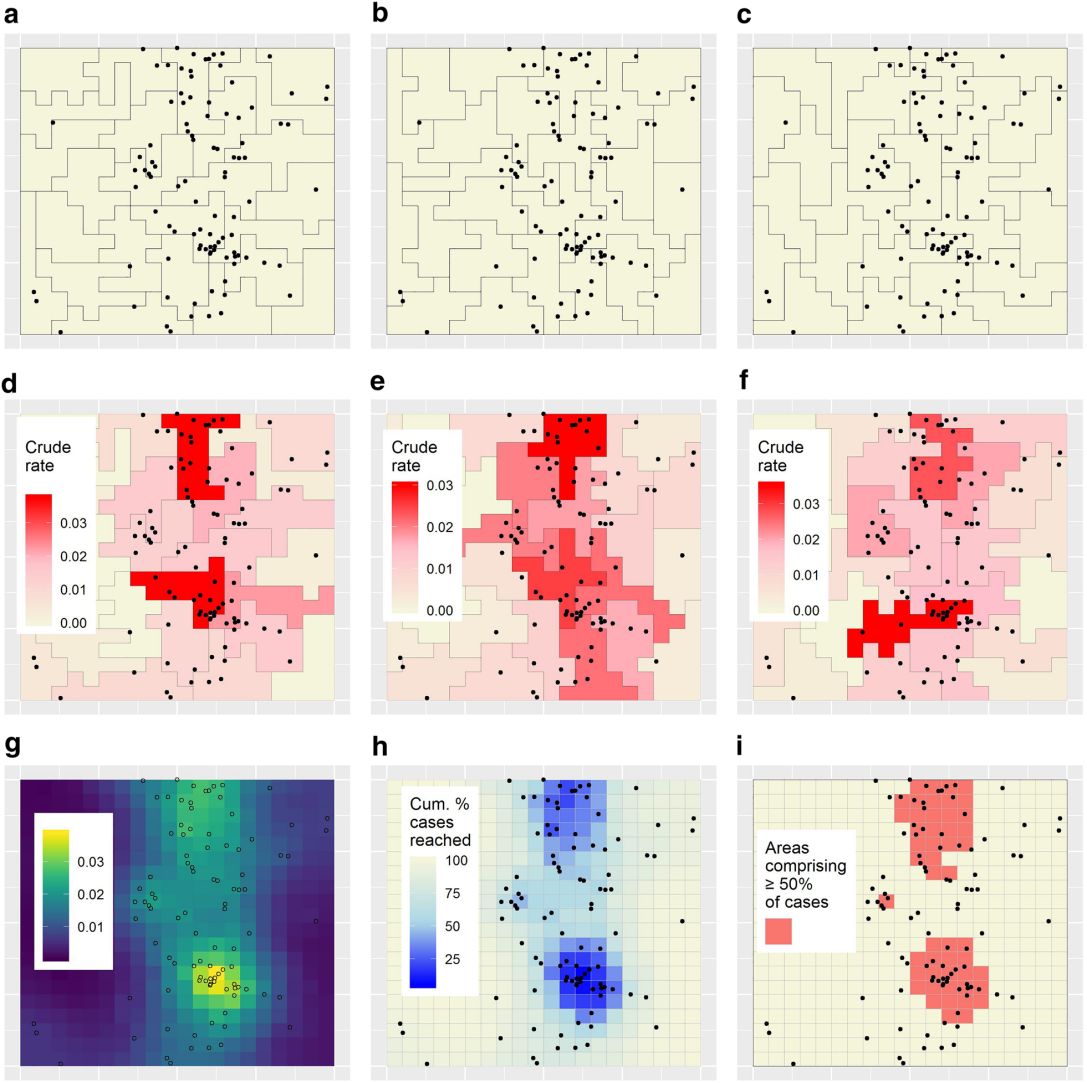
OAM efficiency results for the simulated dataset. **a–c** Three of OAM’s zonations. **d–f** Crude rate disease maps based on **a–c**. **g** Map of population-weighted mean crude rates produced using OAM. **h** Targeting efficiency map associated with **g**. **i** Logistical efficiency map based on a target case percentage of 50%

Figures 4a–c show three of OAM’s 100 zonations, and Figs. 4d–f crude rate maps based on those zonations. Figure 4g shows the minimal-resolution map of population-weighted mean crude rates produced using OAM.

Figure 2a shows the targeting efficiency curve associated with Fig. 4g. The curve lies between the minimal-resolution and single-aggregation curves, illustrating how OAM compromises between those strategies with respect to targeting efficiency. For example, using OAM, 50% of cases could hypothetically be reached through targeting 12.5% of the population (Table 1).

Figure 4h and Fig. 2b show OAM’s targeting efficiency map and logistical efficiency curve, respectively. The latter indicates comparable or somewhat decreased logistical efficiency for OAM as compared to the single-aggregation strategy. For example, using OAM, three discontiguous target regions contain the 12.5% of the population noted above (Table 1). These regions are mapped in Fig. 4i.

### Sensitivity to key parameter values

To investigate OAM’s sensitivity to the number and set of zonations, we created nine additional sets of 100 zonations using AZTool and applied OAM to each. This analysis is described in Additional file 1: ‘Additional results’ (section ‘Sensitivity to key parameter values’). Minimal differences in efficiency were observed between zonation sets and as the number of zonations approached 100, suggesting that the choice of zonation set is relatively immaterial and that 100 zonations is sufficient to mask idiosyncrasies of particular zonations, at least for the simulated dataset examined here.

### OAM as a smoothing technique

Figure 4g has the appearance of being a smooth, albeit relatively grainy surface. This effect derives from the effective creation of spatial smoothing kernels within OAM, characterised by distance decay in the sense that, in the zoning process, nearby units are generally grouped more often than those more distant. Additional file 1: ‘Additional results’ (section ‘Effective smoothing kernels’) illustrates this phenomenon, showing effective smoothing kernels created for two minimal units. Thus, OAM can be characterised as a smoothing technique, with the degree of smoothing governed by the target denominator size and the number of zonations. This is demonstrated in Additional file 1: ‘Additional results’ (section ‘OAM as a smoothing technique’), where we apply OAM to the simulated dataset based on target population sizes of 100, 200, 400, and 800.

### Comparison to existing smoothing techniques

Characterisation of OAM as a smoothing technique suggests comparison to existing smoothing techniques. As an example, we derived a smoothed map of the simulated dataset using a kernel-smoothed spatial density technique implemented in the R package *sparr* [41, 45]. Specifically, we used the *bivariate.density* function in that package. Details of this analysis are provided in Additional file 1: ‘Additional results’ (section ‘Comparison to existing smoothing techniques’). At two different resolutions, maps produced using the *bivariate.density* function and their associated efficiency were markedly similar to those of OAM.

### Global and local zonation dependence

OAM’s incorporation of multiple single-aggregation disease maps uniquely facilitates investigation of the impact of the MAUP’s zonation aspect on such maps. To illustrate, in each of OAM’s component maps, we classify as hotspots those units whose lower crude rate confidence bounds exceed the overall rate of 0.01. Confidence bounds for each unit were derived using the *pois.exact* function implemented in the R package *epitools* [46], based on a confidence level of 64%. However, both the method used to derive confidence bounds and the choice of confidence level are relatively immaterial, since they do not affect the map ultimately produced using OAM or its associated efficiency (see Additional file 1: ‘Additional results’ (section ‘Justification of the zonation-dependence confidence level’)). As a rule of thumb, we suggest choosing a confidence interval such that the hotspot counts derived below range between zero and 100, i.e. the full dynamic range for that quantity.

**Fig. 5:**
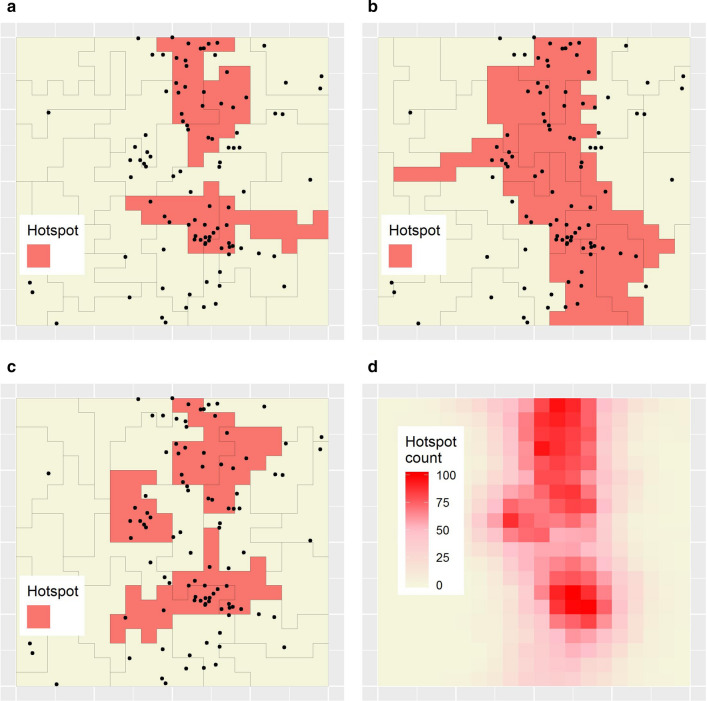
Hotspot analysis results for the simulated dataset.** a–c** Hotspots classified based on Figs. 4d–f. **d** Minimal-resolution map of hotspot counts

Figures 5a–c show hotspots classified based on Figs. 4d–f. Clearly, different hotspots are detected in each map, demonstrating how single-aggregation maps are unreliable for planning purposes. Following [34], but implementing a variation of Eq. (5) in that paper, such differences can be quantified between all pairs of zonations by the average probability that a minimal unit appearing in a hotspot in any given zonation will *not* appear similarly in another. This value measures the global ‘zonation-dependence’ of single-aggregation hotspots. To calculate it, consider that, for minimal units appearing in hotspots in a given zonation ${z}_{i}$, the probability of *not* appearing in a hotspot in a new, different zonation ${z}_{j}$ can be calculated as:

3 $$p_{{j|i}} = ~\frac{{num.~{\kern 1pt}min.~{\kern 1pt} units~in~hotspots~in~zonation~z_{i} ~but~not~zonation~{\kern 1pt} \,z_{j} }}{{num.~{\kern 1pt}~min.~units~in~hotspots~in~zonation~\,z_{i} }}$$

Then, for any given minimal unit appearing in a hotspot in a particular zonation ${z}_{i}$, the average probability ${p}_{.|i}$ of that unit not appearing in a hotspot in another, different zonation may be calculated as the mean of ${p}_{j|i}$ over all $j\ne i$. The mean of values ${p}_{.|i}$ may then be presented with its associated uncertainty represented by the 2.5% and 97.5% quantiles of the distribution of all values ${p}_{.|i}$. Using Eq. (3), we calculated an average zonation-dependence probability of 0.41 (95% quantile interval: 0.34–0.5) for the simulated dataset. This suggests that hotspots classified in single-aggregation disease maps of that dataset are moderately zonation-dependent.

To characterise local zonation dependence, again following [34], we derived a minimal-resolution ‘hotspot count’. This value records the number of zonations in which a given minimal unit appears in a hotspot. It is calculated as:

4 $${HSC}_{m}={\sum }_{p \in P}\left[{HS}_{p}\right]$$

where parameters $m$, $p$, and $P$ are as defined previously; ${HS}_{p}$ is an indicator variable recording whether or not unit $p$ was a hotspot in its zonation; and ${HSC}_{m}$ is the hotspot count derived for unit $m$. Figure 5d shows the hotspot count map for the simulated dataset.

The classification as hotspots (or non-hotspots) of minimal units with hotspot counts equal to zero or 100 is *zonation independent*, i.e. independent of the aggregate-level zonation used. By contrast, the classification of minimal units with hotspot counts between one and 99 is, to varying degrees, *zonation dependent*. Exploring this, we define ‘zonation-dependent negatives’ (ZDNs) to be minimal units classified as hotspots in most (i.e. $\ge$ 80), but not all zonations. Using this threshold, there were 26 ZDNs. Similarly, we define ‘zonation-dependent positives’ (ZDPs) to be minimal units appearing in hotspots in at least one, but relatively few (i.e. $\le$ 20) zonations. Using this threshold, there were 139 ZDPs. Both ZDPs and ZDNs should disturb policymakers, since they effectively represent relatively zonation-independent hotspot or non-hotspot regions that might not be classified as such due to idiosyncrasies of particular single-aggregation disease maps. Illustrating the potential extent of this problem, 41.3% of minimal units were classified as either ZDPs or ZDNs.

Note: the $\ge$ 80 and $\le$ 20 thresholds used above are arbitrary. Depending on the situation, different thresholds might be appropriate; for example, classification of both ZDPs and ZDNs using a threshold of 50 would result in a discussion of regions classified as hotspots (or non-hotspots) in the *majority* of zonations, rather than in *most*.

### Stroke

**Fig. 6:**
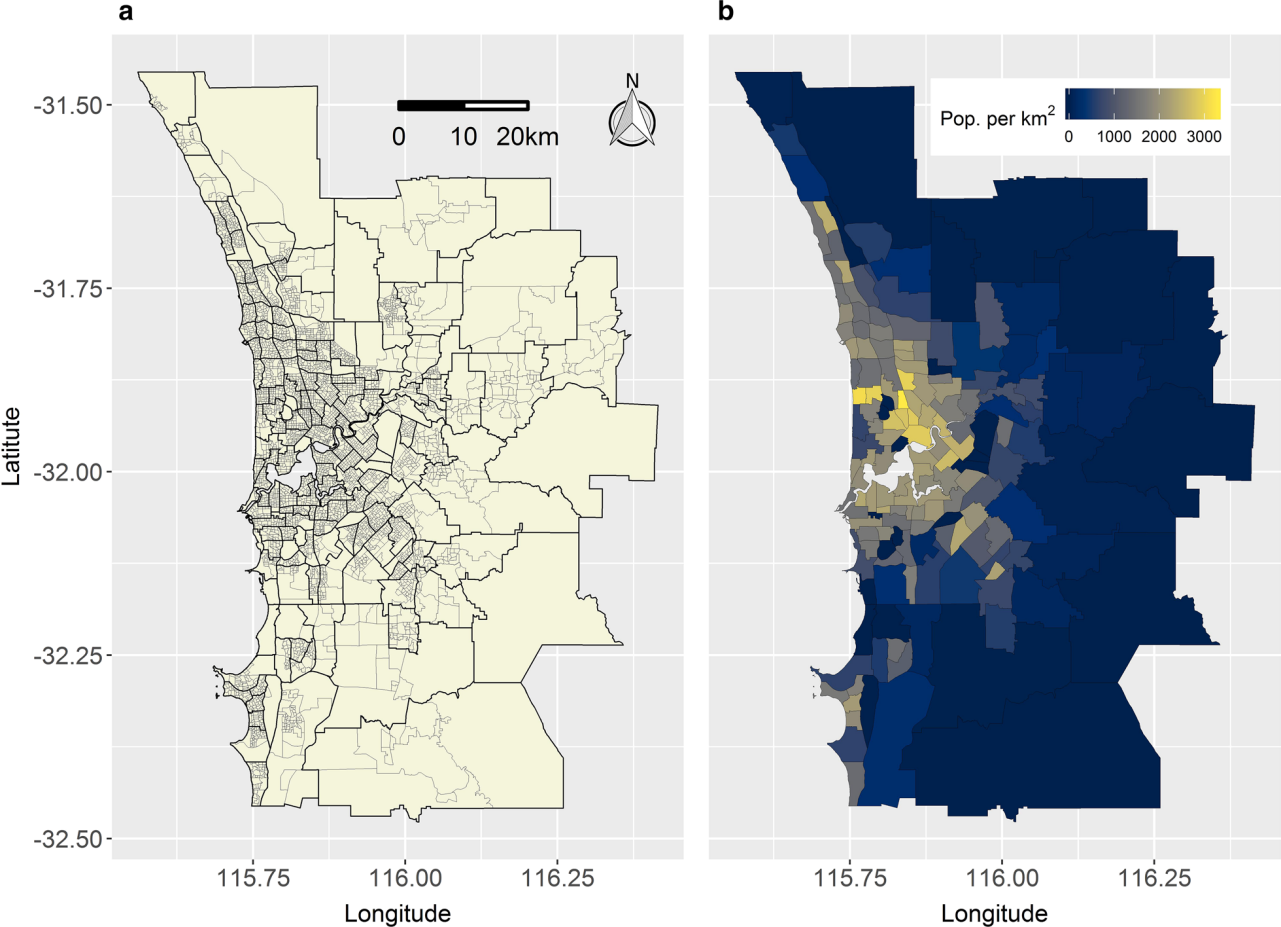
Administrative geography and population density of Perth in 2016. **a** SA1 and SA2 boundaries. **b** SA2-resolution population density

Figure 6a maps the 4,248 SA1s and 164 SA2s in Perth, and Fig. 6b Perth’s 2016 population density by SA2. Perth’s population, which in 2016 was 1.85 million, straddles the Swan and Canning Rivers inland and sprawls north to south along the coastline.

**Fig. 7:**
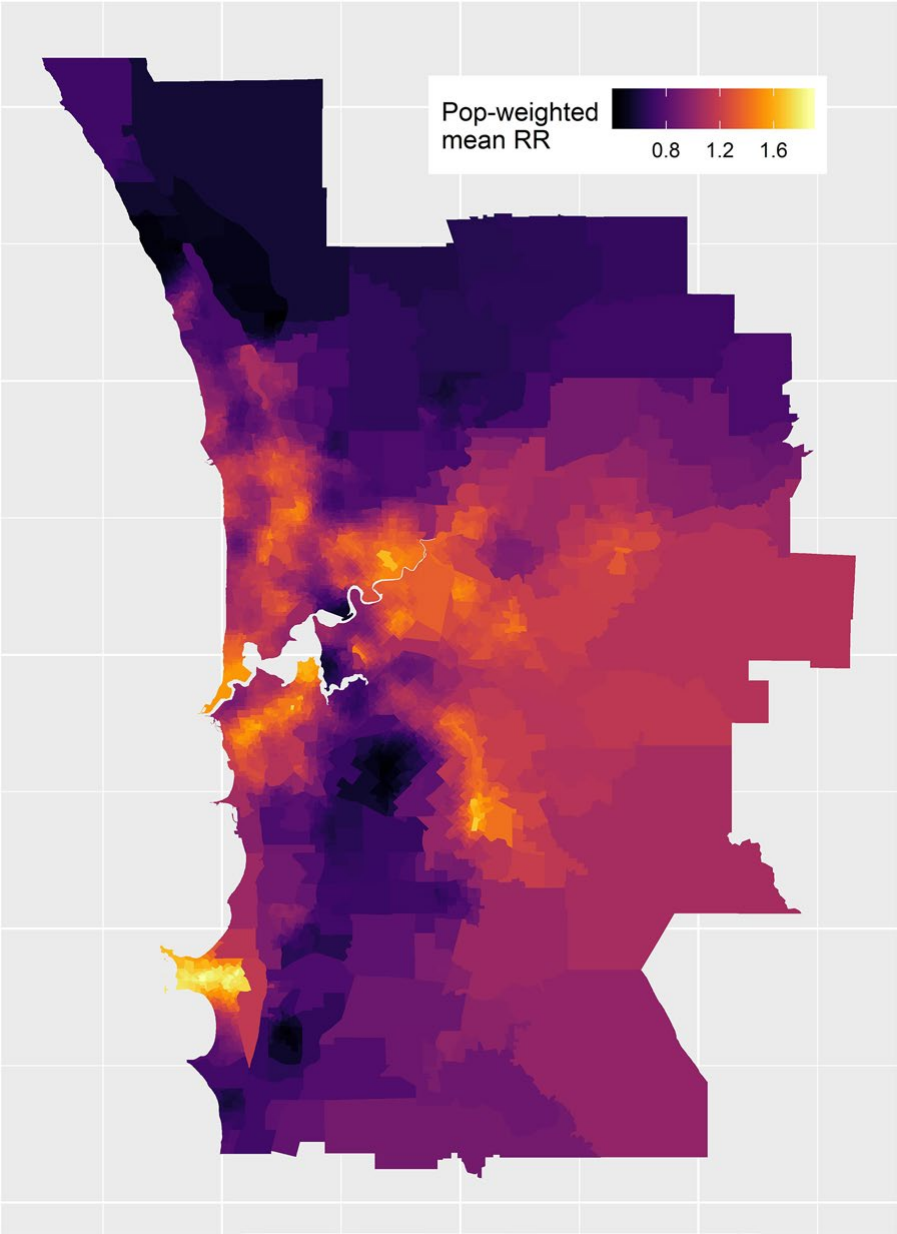
Map of population-weighted mean RRs for stroke

SA1 and SA2 maps of stroke RR are not shown in order to maintain a manageable figure list. However, Fig. 7 shows the map of population-weighted mean RRs produced using OAM. Note: values in this map have been perturbed by a small amount to further protect privacy. Several regions with high weighted RRs are observed, in particular two regions located on the southern coastline and inland in the south-east.

**Fig. 8:**
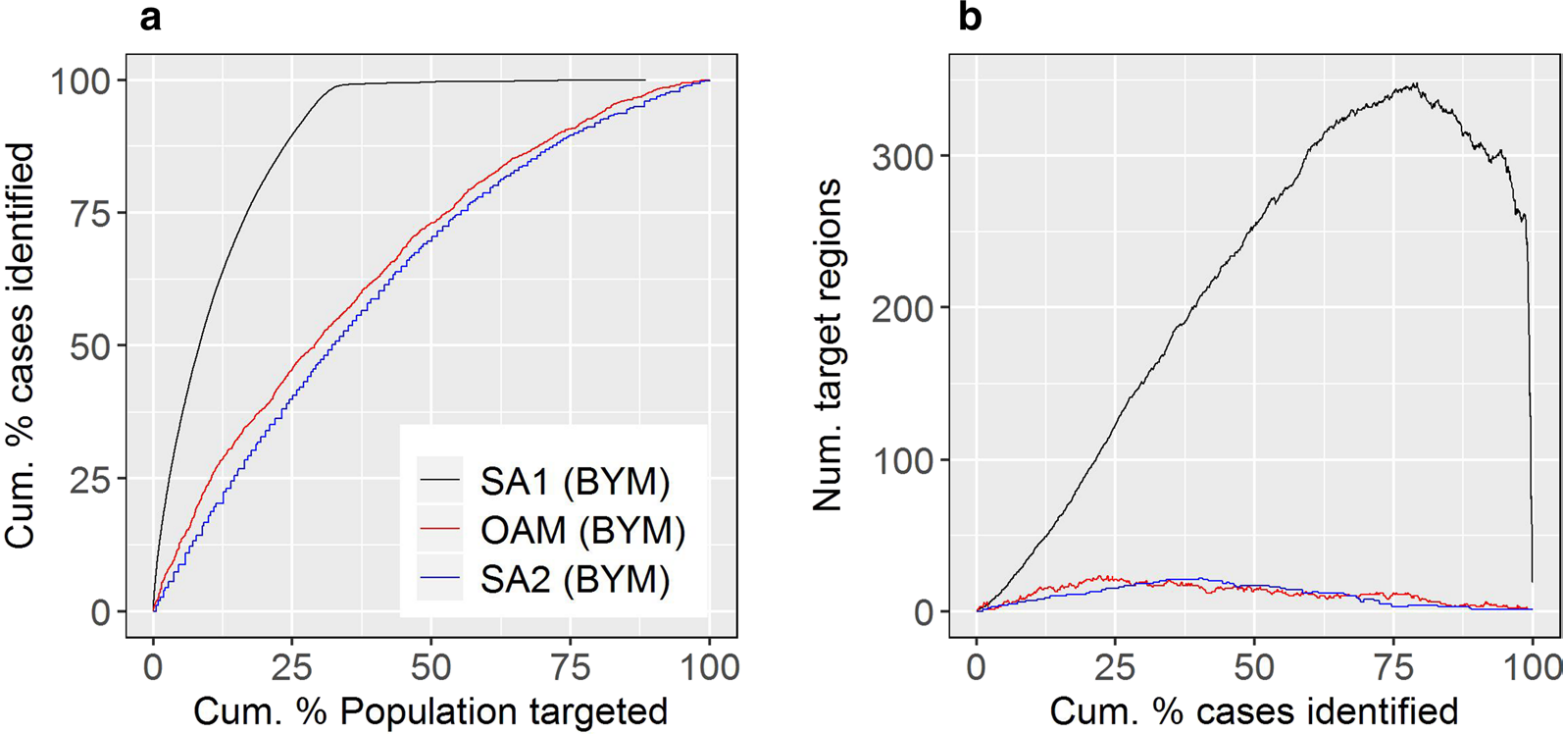
Targeting and logistical efficiency curves for stroke.** a** Targeting efficiency curves. **b** Logistical efficiency curves. Curves shown correspond to maps produced by SA1; SA2; or using OAM

**Fig. 9:**
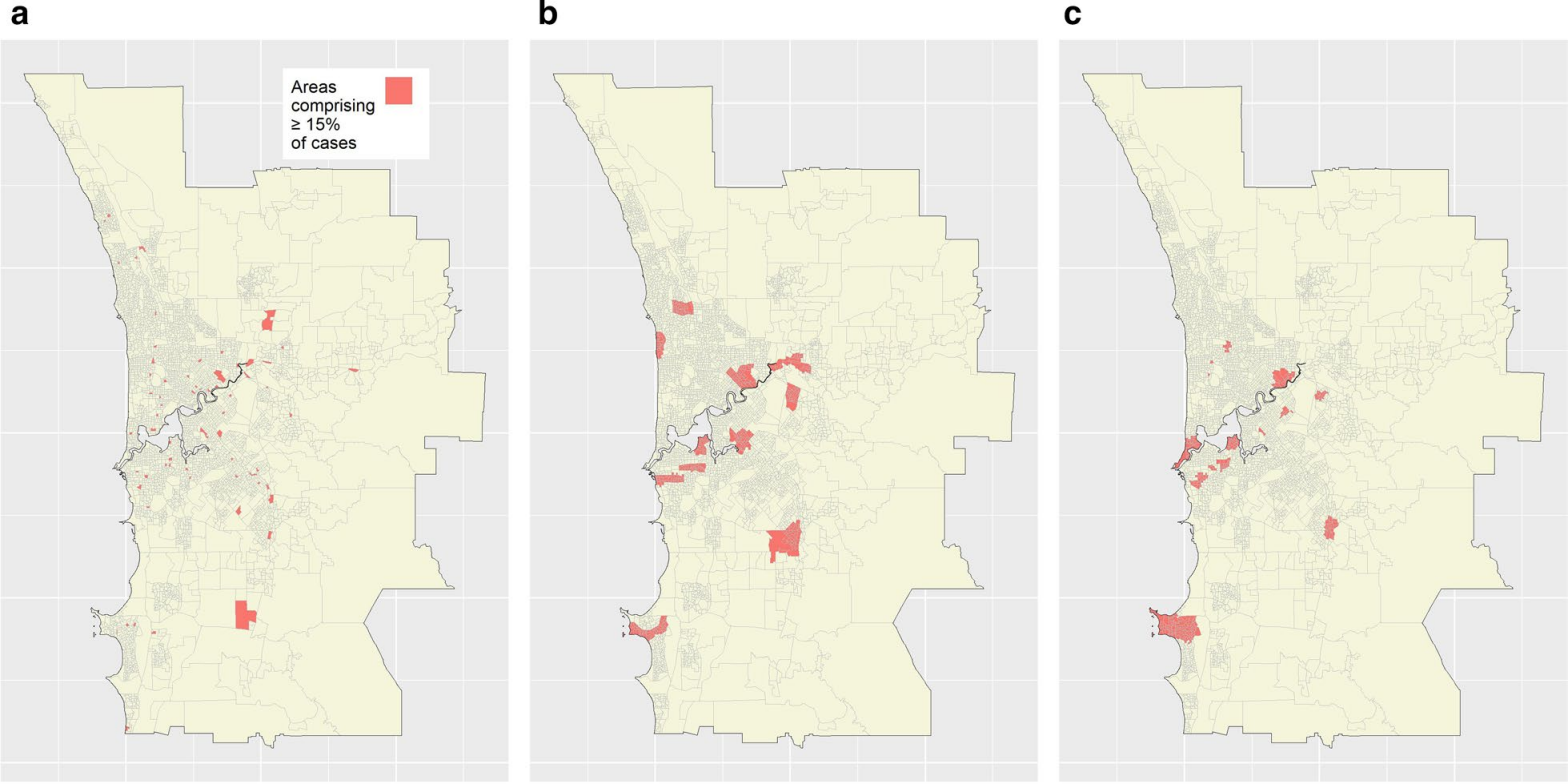
Logistical efficiency maps for stroke based on a target case percentage of 15%. **a** SA1 map. **b** SA2 map. **c** OAM map

Figures 8a-b show the targeting and logistical efficiency curves for the SA1, SA2, and OAM targeting strategies. Consistent with previous results, the SA1 strategy had optimal targeting efficiency but poor logistical efficiency; for example, 15% of cases could hypothetically be reached through targeting 1.4% of the population within 63 discontiguous regions (Table 2). By contrast, the SA2 strategy was characterised by decreased targeting efficiency but increased logistical efficiency compared to the SA1 result (Fig. 8). For example, 15% of cases could be hypothetically be reached through targeting 8.6% of the population within 11 discontiguous regions (Table 2). Finally, OAM balanced targeting and logistical efficiency (Fig. 8); using OAM, to hypothetically reach 15% of cases, 5.9% of the population would need to be targeted within 15 discontiguous regions (Table 2). Figures 9a–c show the logistical efficiency maps corresponding to the target case percentage of 15%, for the three strategies.

**Table 2:** Exact efficiency data for stroke

Mapping method	Cum. % of population targeted	Number of target regions
SA1	1.4	63
OAM	5.9	15
SA2	8.6	11

Shown are cumulative percentage of population targeted and number of target regions values associated with a target case percentage of 15%, for the SA1, SA2, and OAM targeting strategies.

**Fig. 10:**
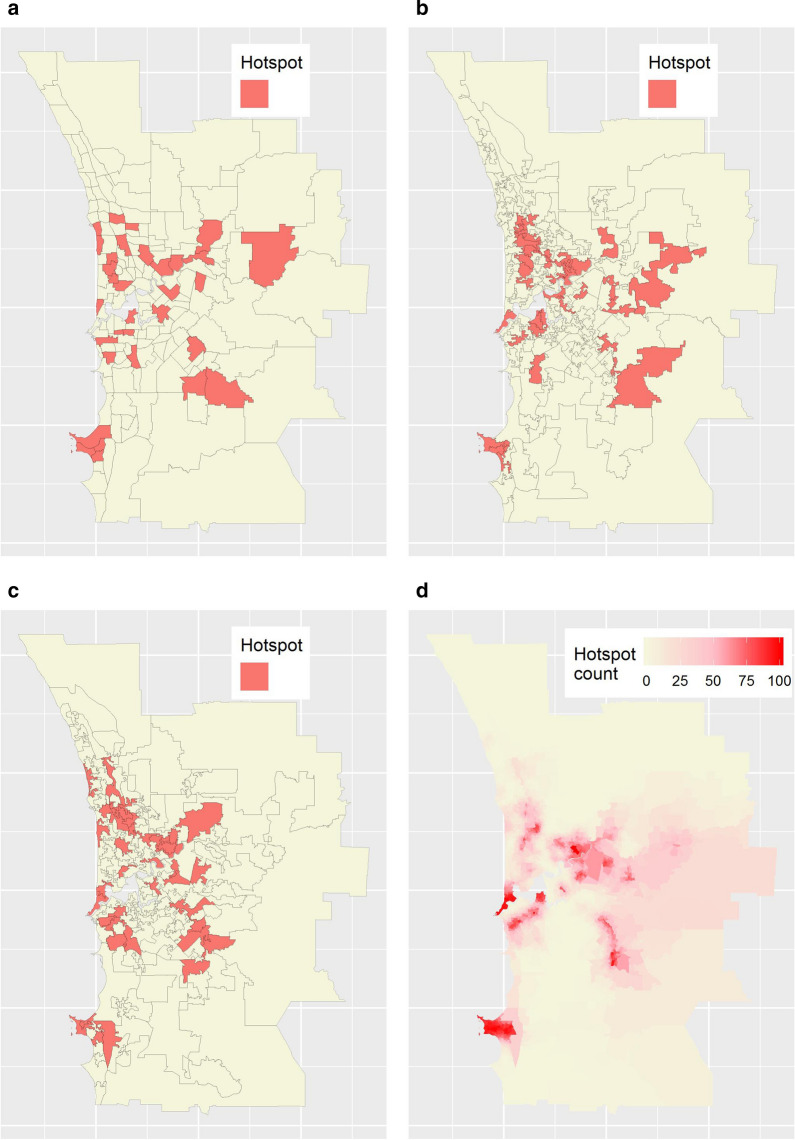
Hotspot analysis results for stroke. **a** SA2 hotspots. **b**, **c** Hotspots based on two of OAM’s zonations. **d** SA1-resolution hotspot counts

Figures 10a–c show hotspots classified among SA2s and in two of OAM’s zonations. Here, following the rule of thumb proposed previously, hotspots were defined to be those areas whose lower 81% credible interval bounds exceeded 1. As for the simulated dataset, different hotspots are detected in each map in Figs. 10a–c, reinforcing the previous conclusion that single-aggregation disease maps are unreliable for planning purposes. We calculated a global probability of zonation-dependence of 0.52 (95% quantile interval: 0.48–0.56); this suggests that, on average, a hotspot in a given single-aggregation disease map of stroke will, as often as not, *not* be similarly classified in a different map of the same data.

Figure 10d maps the SA1 hotspot counts for stroke. Applying the same thresholds used previously (i.e. $\ge$ 80 and $\le$ 20), we classified 133 stroke ZDNs and 1,823 stroke ZDPs. Together, these regions comprised almost half (46%) of the entire study area.

Finally, to explore the impact on efficiency of implementing a complex model versus no model within OAM, Additional file 1: ‘Additional results’ (section ‘Crude rate analysis of stroke’) describes a crude rate analysis of stroke. As might be expected, regardless of the targeting strategy (i.e. SA1, SA2, or OAM), the crude rate analysis was characterised by increased targeting efficiency but decreased logistical efficiency compared to the BYM model analysis. This is due to the additional smoothing of the underlying data that occurs when using the BYM model.

## Discussion

We have introduced OAM and demonstrated its utility in efficiently mapping the geographical distribution of stroke in Perth in 2016. OAM identified several regions with high risk of stroke, in particular two regions located on Perth’s southern coastline and inland in the south-east. In planning interventions, these regions might be considered alongside information such as proximity to specialist stroke units or ambulance depots. An example intervention might be the deployment of mobile stroke units, such as has occurred in some cities in recent years [47]. However, it is not within scope for this paper to advocate or evaluate the feasibility of any particular intervention.

Perhaps most critically, we have demonstrated how the classification of hotspots (or non-hotspots) in single-aggregation disease maps may depend on the particular aggregate-level zonation used. Therefore, no result in any single-aggregation map is reliable. OAM constitutes a novel framework to quantify this phenomenon and describe its local effects. However, we note the extent of the problem is likely to vary by case. Where disease clusters are larger than the chosen mapping units, agreement between single-aggregation maps based on different, but similarly sized units is likely to be high. An example of this might be the mapping of under-5 mortality in sub-Saharan Africa using a grid of 5 × 5 km units [48]. In that study, regions with high mortality rates were generally large compared to the chosen units; therefore, it is likely that different configurations of the chosen grid would have identified approximately similar high-rate regions. By contrast, where disease clusters are smaller than the chosen units, agreement between single-aggregation maps based on alternative configurations of those units is likely to be lower. An example of this might be the previously-cited study of cholera in sub-Saharan Africa [8], where many of the classified hotspots were relatively punctate. Therefore, it is likely that different configurations of the grid of 20 × 20 km units would have identified quite different hotspots. Future work should investigate potential differences in zonation dependence between datasets, as alluded to above.

Further, we have demonstrated how the impact of the MAUP on single-aggregation disease maps manifests in substantially reduced targeting efficiency due to prioritisation of logistical efficiency. This should disturb policymakers, given the generally scarce nature of healthcare resources. OAM’s second function as a smoothing technique represents a solution, with maps produced using OAM balancing the two aspects of efficiency. However, in demonstrating this, we have shown how OAM’s output is strikingly similar to that of at least one existing smoothing technique applied to minimal-resolution data. Therefore, beyond the fact that such techniques have not previously considered efficiency as defined here, below we outline the key features differentiating them from OAM.

Most notably, OAM facilitates derivation of smoothed, minimal-resolution estimates without directly modelling minimal-resolution data. This is achieved through models being fitted only to aggregate-level data, a generally less prohibitive task than would be their direct application to minimal-resolution data. Thus, more complex models than could otherwise be fitted can be applied within OAM; for example models including covariates or fully Bayesian models. To our knowledge, this feature is unique in the literature, with the exception of certain interpolation techniques that allow for derivation of estimates below the resolution at which data are available (e.g. see [49]). However, unlike OAM, such techniques are potentially undermined by the ecological fallacy, a problem related to, but distinct from, the MAUP.

Where both OAM and an alternative smoothing technique could be implemented, it might still be more advantageous to implement OAM, for these reasons: first, OAM uniquely accounts for edge effects. In contrast to existing smoothing techniques, which account for edge effects through implementing various corrections (e.g. see [45]), OAM does so through its effective smoothing kernels naturally adapting to the study area boundary and other borders. Second, OAM could be extended to model spatiotemporal data. This might involve simply examining its zonations at multiple time points. By contrast, not all existing smoothing techniques can model spatiotemporal data. Third, OAM stabilises population sizes between spatial units. Additional file 1: ‘Additional results’ (section ‘Stabilisation of population sizes’) demonstrates this, showing the distribution of population sizes among SA1s, SA2s, and units within OAM’s zonations in the stroke analysis. In this attribute, OAM is unique from most existing smoothing techniques, with the exception of some kernel-smoothing approaches that implement adaptive bandwidths (e.g. see [50]). However, those techniques are often limited in their ability to additionally examine covariates or spatiotemporal data. Further, it has been noted that they might be computationally expensive even for moderately-sized datasets [51]. Finally, OAM protects patient privacy, since the process of combining information from multiple single-aggregation disease maps constitutes a geographical encryption key. To back-calculate data input into OAM would require: (i) extracting the values displayed in a given map; (ii) reversing their perturbation; and (iii) identifying a set of zonations and distribution of cases which, when modelled, exactly reproduce the unperturbed values. These steps constitute a complex deconvolution problem, almost certainly without a unique solution. Accordingly, some minimal-resolution values could be reported, since this would negligibly reduce the problem’s complexity.

Importantly, we note that the impact of the MAUP described, quantified and overcome by OAM is that of its zonation aspect arising due to aggregation beyond the minimal resolution. OAM does not overcome the corresponding impact of the MAUP’s scale aspect; rather, we have simply demonstrated that an increased scale of aggregation within OAM results in increased smoothing. This is unavoidable, and also characterises the output of other smoothing techniques. In addition, OAM does not assess the impact of the MAUP as manifesting due to aggregation by the minimal unit in the first place. That impact is also unavoidable when producing a disease map, since to do so the study area must be discretised in some way. Within OAM, this occurs when data are aggregated by the minimal units. By comparison, kernel-smoothing algorithms usually display information by a fine-resolution grid. As another example, spatial point process models often approximate a continuous spatial intensity surface at a fine, grid-based resolution, or in a more complex manner through applying basis functions across a triangulated mesh [42]. As a third possibility, discretisation might occur as a final step, i.e. post-modelling, purely for the purpose of producing a map.

It could be argued that the examination of multiple different zonations within OAM ignores the inherent importance or meaningfulness of pre-defined administrative areas. This view is correct in certain aspects; for example, funding to address disease is often distributed based on local government areas, health districts, or hospital catchments. Further, certain interventions are best targeted by administrative boundaries. For example, at the time of writing, some regions in Victoria, Australia had recently re-entered ‘lockdown’ to address a second wave of COVID-19 [15]. The impending lockdown was communicated to residents through reference to postcode boundaries, and it is likely that any other approach would have caused confusion. However, this reality should not preclude the situation where interventions to address disease are targeted to regions potentially overlapping different administrative units. Since intervention resources are invariably scarce, as a general rule, disease should be mapped as it is distributed, and funding or other intervention measures adapted to this distribution to most effectively and efficiently address it, not the other way around.

The scale of aggregation within OAM parallels the choice of a smoothing bandwidth in some other smoothing techniques. There is a rich literature devoted to evaluating various data-based or theoretical techniques of bandwidth selection for such methods (e.g. see [52]). Future work might investigate application of these techniques within OAM. In the meantime, we suggest users choose an appropriate scale of aggregation guided by the characteristics of a planned intervention. For example, a relatively fine scale might be appropriate when planning a highly localised intervention, since this will result in delineation of increasingly punctate and discontiguous target regions. However, such an approach will potentially be limited by computational constraints and the relative rareness of the disease being examined (rare conditions will generally require a greater degree of aggregation).

There is potential to extend OAM to incorporate statistical measures of uncertainty (e.g. confidence intervals) for its map output. Since this functionality is not currently included, OAM cannot be used to statistically classify hotspots, such as is often desired in practice to characterise inequality between geographical regions. This was intentional, since our focus has been to demonstrate OAM’s utility in delineating target regions characterised by their efficiency, and compare this efficiency to that of single-aggregation maps. While future work might extend OAM to enable classification of hotspots, it would not be sensible to quantify uncertainty in OAM’s efficiency output, since, by definition, that output is directly associated with a given observed dataset.

While AZTool is a useful, free software, it is not an essential component of OAM; OAM’s zonations could be created using any one of several available zonation tools, many of them automated. Such tools have become increasingly available in recent years, though they are not always free. Alternatively, custom computer code could be written. Future work might investigate the potential impact of implementing OAM using zonations created using different software.

Our presentation of OAM in the first instance as applied to a point location dataset generalises our findings beyond the specific set of administrative units in the stroke application. Essentially, it is unimportant whether the minimal and aggregate-level units are polygons (e.g. administrative units) or grid cells. For the simulation, the minimal units were grid cells while the aggregate-level units were polygons. By contrast, for stroke, both the minimal and aggregate-level units were administrative units. As a third possibility, only aggregate-level information might be available, but for multiple zonations. In that case, a minimal-resolution value could still be derived using OAM, simply through implementing its fourth and fifth steps. Future work might investigate the exact way in which such a procedure might be implemented.

As a final point, we consider another disease mapping method commonly applied to both point location and areal disease data: spatial scans [53]. As an example, we consider the popular method SaTScan [54, 55]. Within SaTScan, scanning windows of different sizes that are usually circular are evaluated across a given geographical study area, with the aim of identifying one or more ‘likely’ clusters. Our interest is to compare such output to that of OAM in the context of efficiency. As such, we note that spatial scans differ from the smoothing techniques described above in that they cannot output minimal-resolution values; rather, values associated with each window are output. Thus, it is not clear how such output might be used to calculate targeting and logistical efficiency. Key to doing so would be effective management of the overlapping windows. Future work might usefully investigate different strategies to achieve this.

## Conclusions

In conclusion, we have demonstrated how pronounced inefficiencies could result from reliance on single-aggregation disease maps to guide distribution of healthcare resources. Routine implementation of OAM can help prevent this. We hope that this paper will be a catalyst for increased awareness and acknowledgement of the MAUP in disease mapping and beyond. As a first step, studies examining areal data, both within and beyond the context of disease mapping, should routinely acknowledge the MAUP as a limiting feature whenever it is impossible to explicitly address its impact.

## Supplementary information

**Additional file 1.** Additional results.

## Data Availability

The authors declare that data supporting the findings of this study are available within the paper. Population data for Perth are freely available online. Hospitalisation data for Perth are confidential and available by application to the Department of Health Western Australia, Data Linkage Branch.

## Abbreviations

ABS: Australian Bureau of Statistics
HMDC: Hospital Morbidity Data Collection
MAUP: Modifiable areal unit problem
OAM: Overlay aggregation method
SA1-4: Statistical Areas Levels 1-4
WA: Western Australia
ZDP: Zonation-dependent positive
ZDN: Zonation-dependent negative
