# Supplementary material for: Overcoming inefficiencies arising due to the impact of the modifiable areal unit problem on single-aggregation disease maps
Source: Int J Health Geogr. 2020 Oct 3;19:40. doi: 10.1186/s12942-020-00236-y (PMC7532343; doi:10.1186/s12942-020-00236-y)
Supplement: Supplementary file 1 — Additional file 1. Additional results. [file 12942_2020_236_MOESM1_ESM.docx]

**Additional File 1: Additional results**

**Contents**

**Sensitivity to key parameter values**

**Effective smoothing kernels**

**OAM as a smoothing technique**

**Comparison to existing smoothing techniques**

**Justification of the zonation-dependence confidence level**

**Crude rate analysis of stroke**

**Supplementary references**

**Stabilisation of population sizes**

**Sensitivity to key parameter values**

Supplementary Fig. S1a shows targeting efficiency curves obtained through applying OAM to the simulated dataset using each of ten different zonation sets. Minimal differences between the curves suggest that the particular choice of zonation set is relatively immaterial to OAM’s final output. A possible reason for this is that the *number* of zonations created is large enough that idiosyncrasies of particular zonations within each set do not unduly impact. Supporting this theory, Supplementary Fig. S1b shows, for each zonation set, the mean absolute percentage difference in minimal-resolution population-weighted mean crude rates output by OAM, associated with incremental increases in the number of zonations. For a given zonation set, this value is calculated as:

|  | $X_{i}=\frac{1}{M_{n}}\sum_{m \in M} \left\vert\frac{v_{m\vert i}-v_{m\vert i-1}}{v_{m\vert i-1}} \right\vert$ | (S1) |
| --- | --- | --- |

where $v_{m|i}$ and $v_{m|i-1}$ are the population-weighted mean crude rates for minimal unit $m\in M$, calculated using Equation (2) in the main text and using $i$ and $i-1$ zonations, respectively; and $M$ is the set of $M_{n}$ minimal units ($M_{n}$ = 400 for the simulated dataset). To create Supplementary Fig. S1b, we implemented Equation (S1) for $i\in2\ldots100$. A gradual decrease in the mean absolute percentage difference is observed as the number of zonations increases, with minimal variation in the rate and magnitude of the decrease between sets. Further, for all curves, a plateau is reached where the percentage difference equals approximately zero, which occurs when $i=100$. This suggests that use of 100 zonations is sufficient to produce approximately reproducible results, at least for the simulated dataset examined here.

**Supplementary Fig. S1.** Sensitivity analysis results for OAM applied to the simulated dataset. **a** Targeting efficiency curves. **b** Mean absolute percentage change in population-weighted mean crude rate curves. Different curves in each panel correspond to different zonation sets.


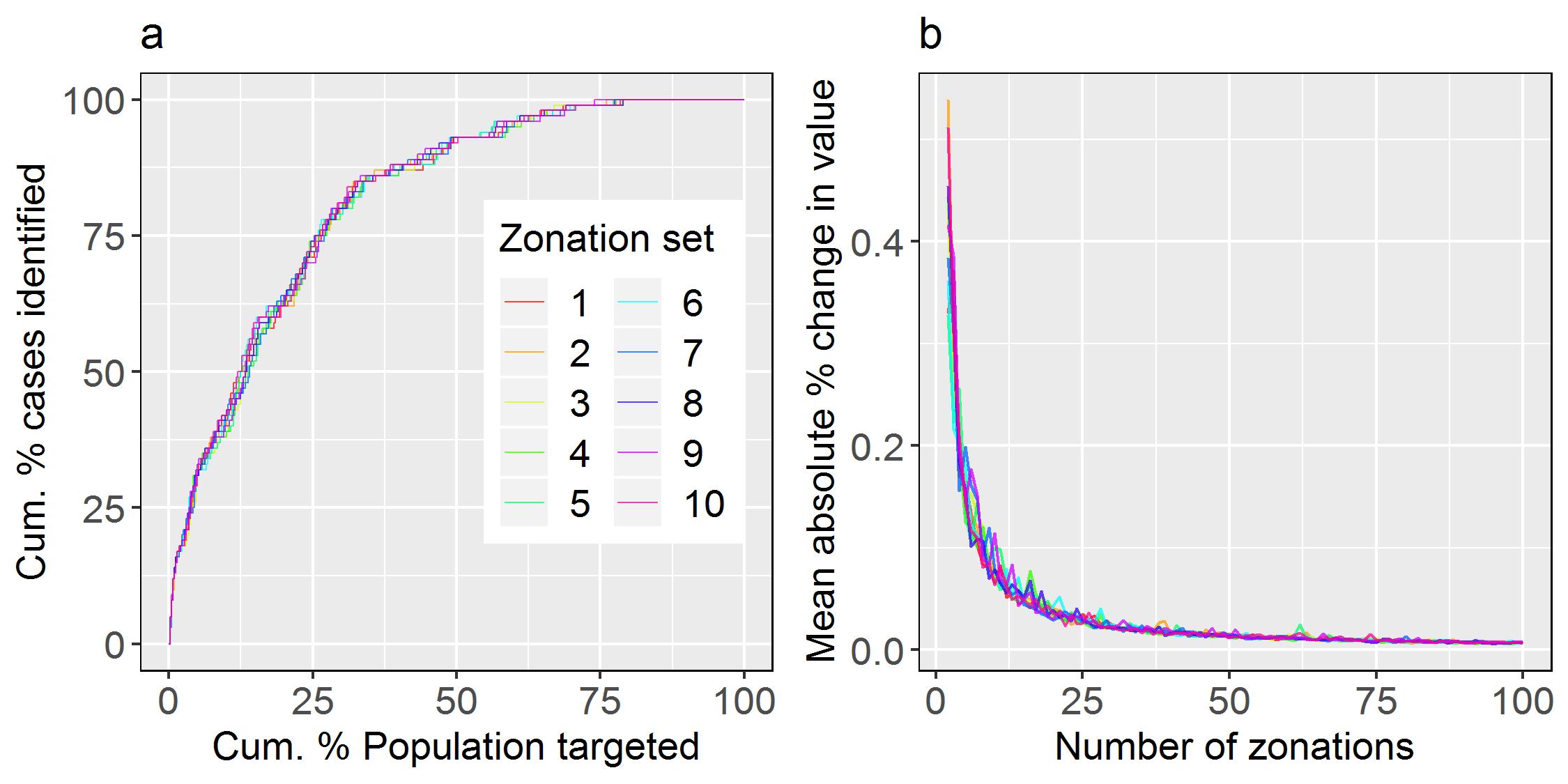


**Effective smoothing kernels**

Supplementary Figs. S2a-c show three of the aggregate-level units comprising a particular ‘source’ minimal unit near the centre of the simulation study area. These units are from OAM’s first three zonations (see Figs. 4a-c; main text). When all 100 of the aggregate-level units comprising the indicated source unit are overlaid, the result is the effective smoothing kernel shown in Supplementary Fig. S2d. In that plot, minimal units are coloured gradually more red according to the number of times they are grouped with the source unit.

The effective smoothing kernel in Supplementary Fig. S2d is approximately circular, but this will not always be the case. Aggregate-level units near the study area boundary or other internal boundaries (e.g. those formed by natural features such as lakes or rivers), and effective smoothing kernels based on those units, will naturally adapt to the shape of those boundaries. Supplementary Fig. S3 illustrates this phenomenon, replicating Supplementary Fig. S2 for a source minimal unit located on the left border of the study area.

**Supplementary Fig. S2.** Illustration of an effective smoothing kernel. **a-c** Aggregate-level units (shaded pink) comprising an exemplar source minimal unit, from three of OAM’s zonations. **d** The effective smoothing kernel for the source unit. In **d**, the source unit itself has a value of 100.

**
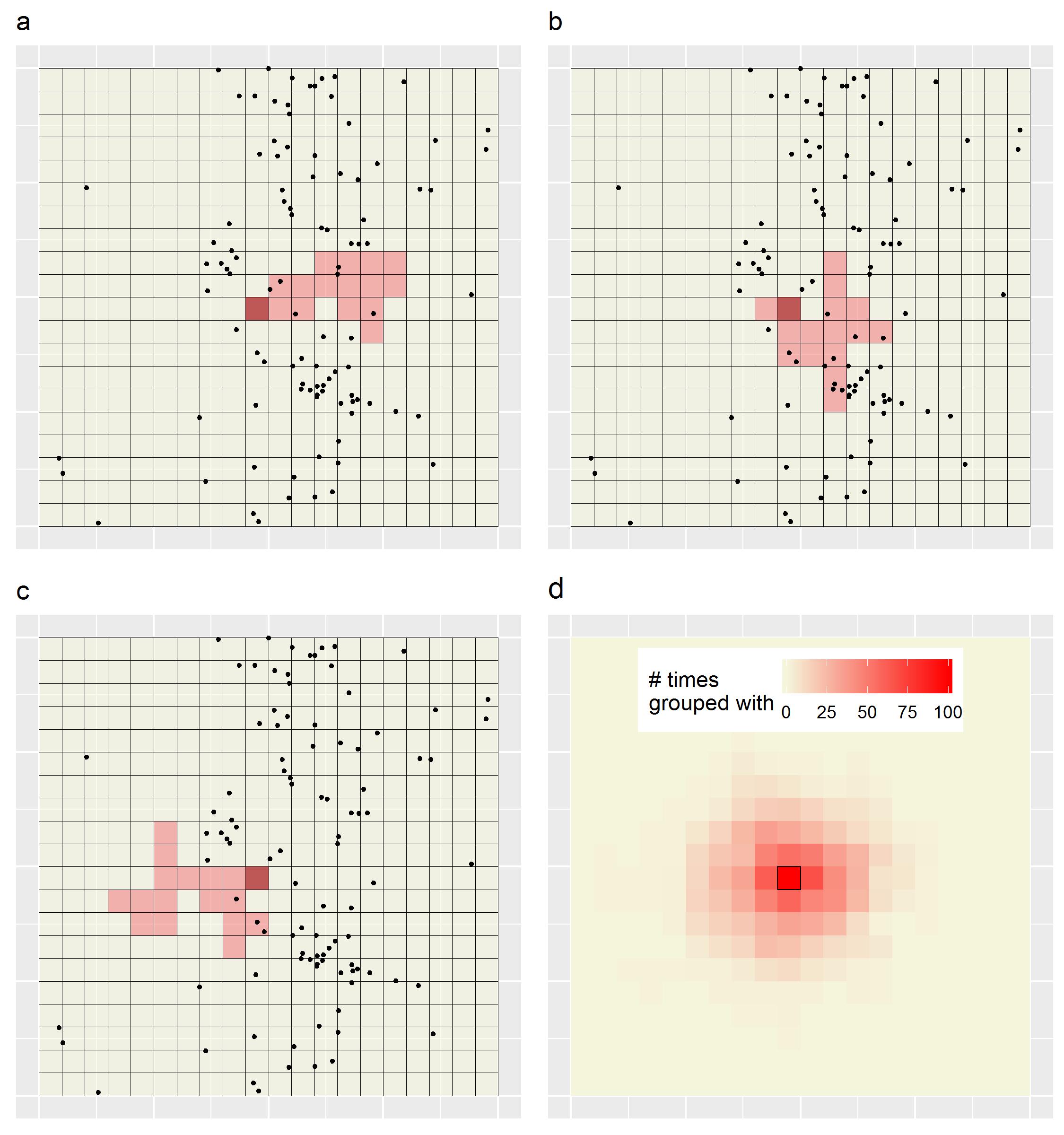
**

**Supplementary Fig. S3.** Reproduction of Additional File 1, Supplementary Fig. S2 for a different source minimal unit.

**
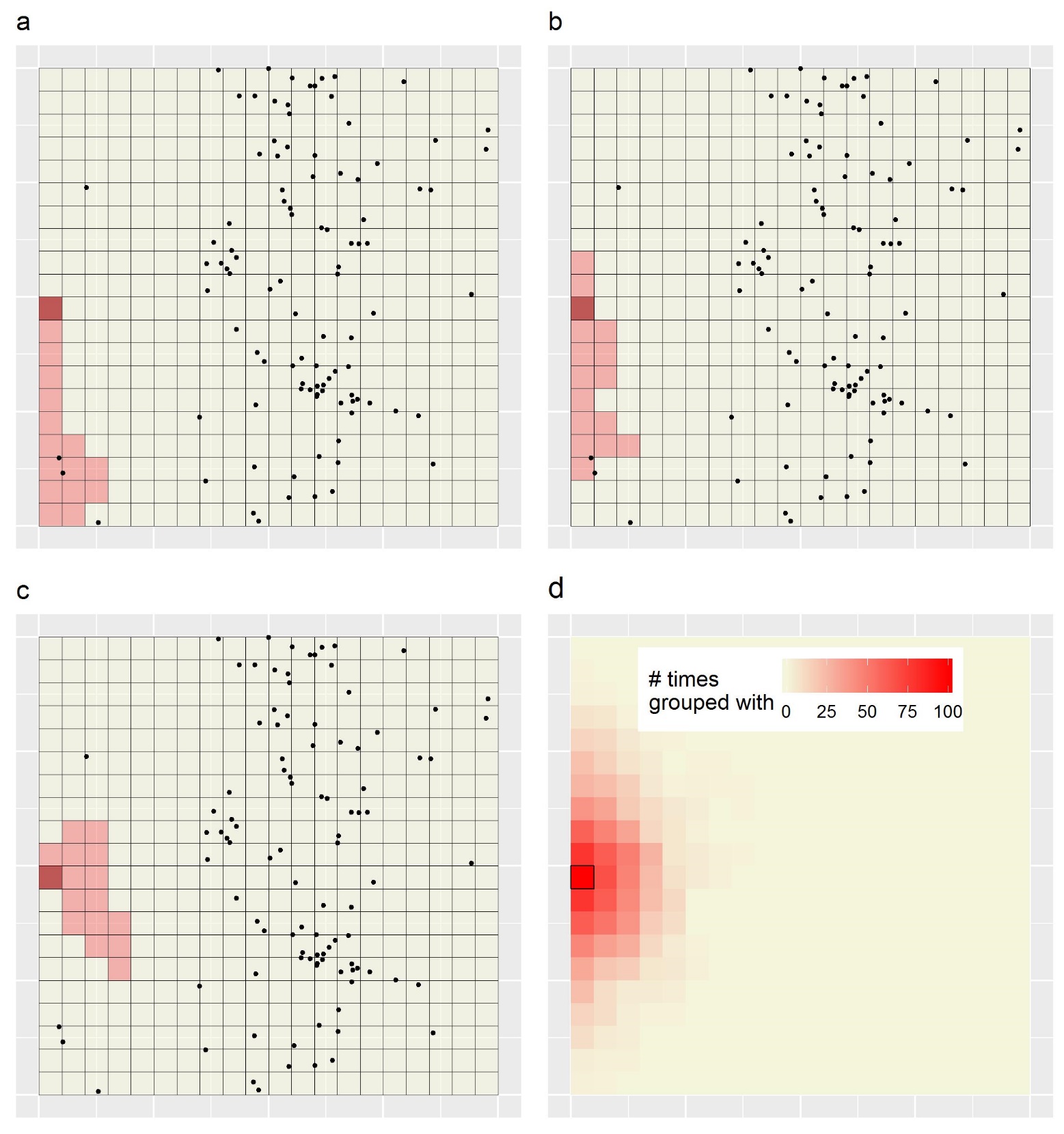
**

**OAM as a smoothing technique**

Supplementary Figs. S4a-d show maps produced using OAM as applied to the simulated dataset, based on target population sizes of 100, 200, 400, and 800. Corresponding minimum threshold population sizes were 75, 150, 300, and 600, respectively. Note: Supplementary Fig. S4c reproduces Fig. 4g in the main text. Together, these maps demonstrate how the degree of smoothing in OAM’s output is proportional to the specified target denominator size.

The result of decreased smoothing is increased targeting efficiency but decreased efficiency as the minimal-resolution strategy (i.e. no smoothing) is approached. Demonstrating this, Supplementary Figs. S5a-b show the targeting efficiency and logistical efficiency curves associated Supplementary Figs. 4a-d. The corresponding minimal-resolution curves (Fig. 2; main text) are reproduced for reference. Note: the curves associated with a target population size of 400 reproduce OAM’s curves in Fig. 2 of the main text.

**Supplementary Fig. S4.** Maps produced using OAM based on different target population sizes. **a-d** Maps based on target population sizes of 100; 200; 400; and 800, respectively. In **a-d**, values shown are population-weighted mean crude rates.


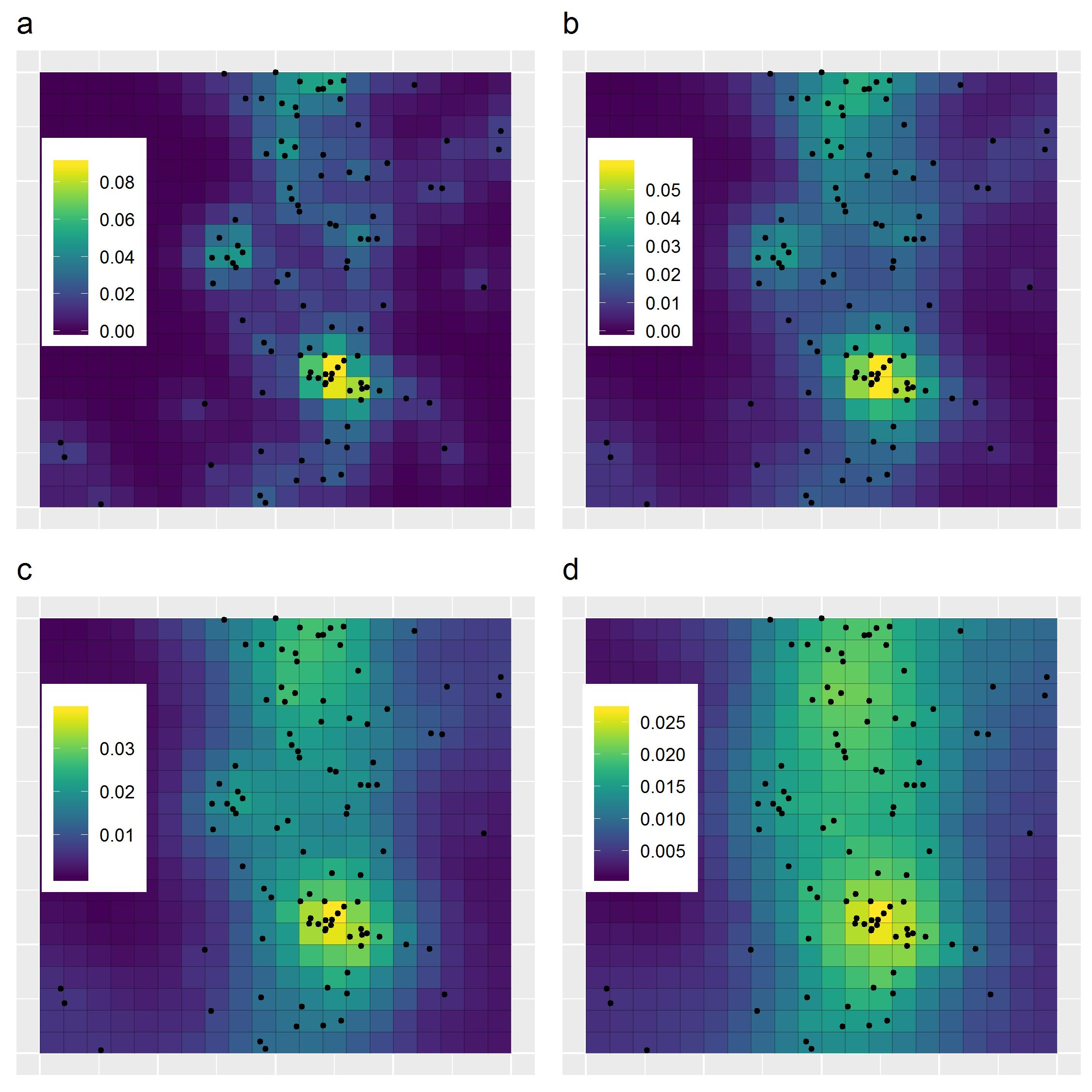


**Supplementary Fig. S5.** Targeting and logistical efficiency curves associated with Supplementary Figs. S4a-d and the minimal-resolution strategy. **a** Targeting efficiency curves. **b** Logistical efficiency curves.


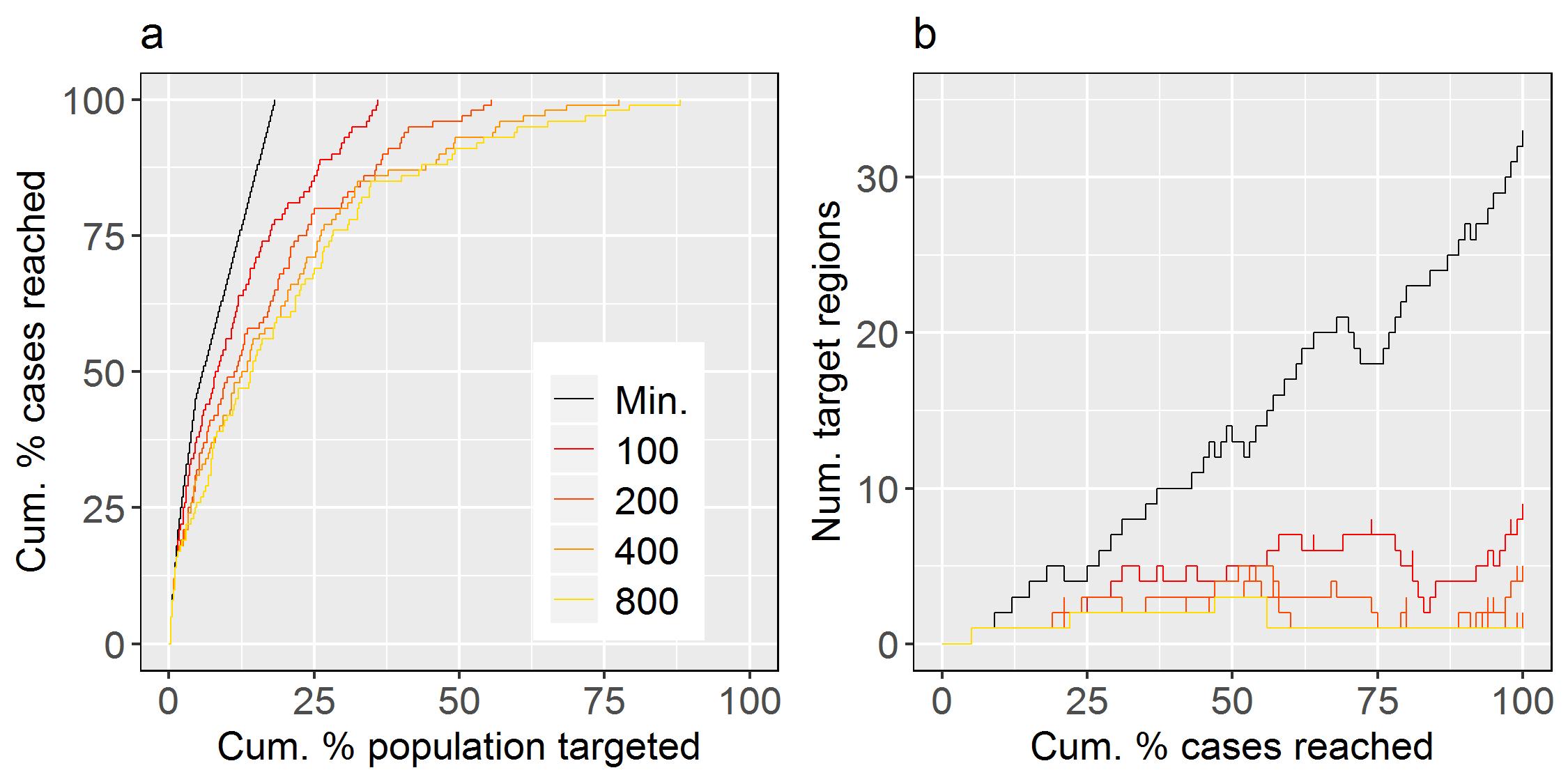


**Comparison to existing smoothing techniques**

To facilitate comparison with OAM’s output, we parameterised the *bivariate.density* function with a fixed bandwidth of 0.08 and a 20 x 20 unit resolution. Note: the result is a smoothed *density* map (i.e. a map of disease rates per unit area), whereas OAM has examined crude rates. However, their output is comparable in this simplistic scenario, since we define the population size and area of each minimal unit to be equal.

Supplementary Fig. S6a reproduces Fig. 4g (main text) and Supplementary Fig. S6b shows the map produced using the *bivariate.density* function. The maps are similar in appearance, which is expected given we specifically parameterised the *bivariate.density* function to achieve this. Their targeting and logistical efficiency curves were also similar, as demonstrated in Supplementary Figs. S7a-b. In those figures, the corresponding minimal-resolution and single-aggregation curves (Fig. 2; main text) are shown for reference.

To further illustrate, we reapplied both OAM and the *bivariate.density* function based on the same target population size and bandwidth but with a resolution of 100 x 100. Supplementary Figs. 6c-d show the resulting maps, which both more closely approximate a continuous surface. This supports characterisation of OAM as a smoothing technique.

**Supplementary Fig. S6.** Maps produced using OAM and kernel-smoothed technique. **a,c** OAM-smoothed maps. **b,d** Smoothed maps obtained using a kernel-smoothed spatial density technique. Maps in **a,b** are based on a 20 x 20 unit resolution. Maps in **c,d** are based on a 100 x 100 unit resolution.


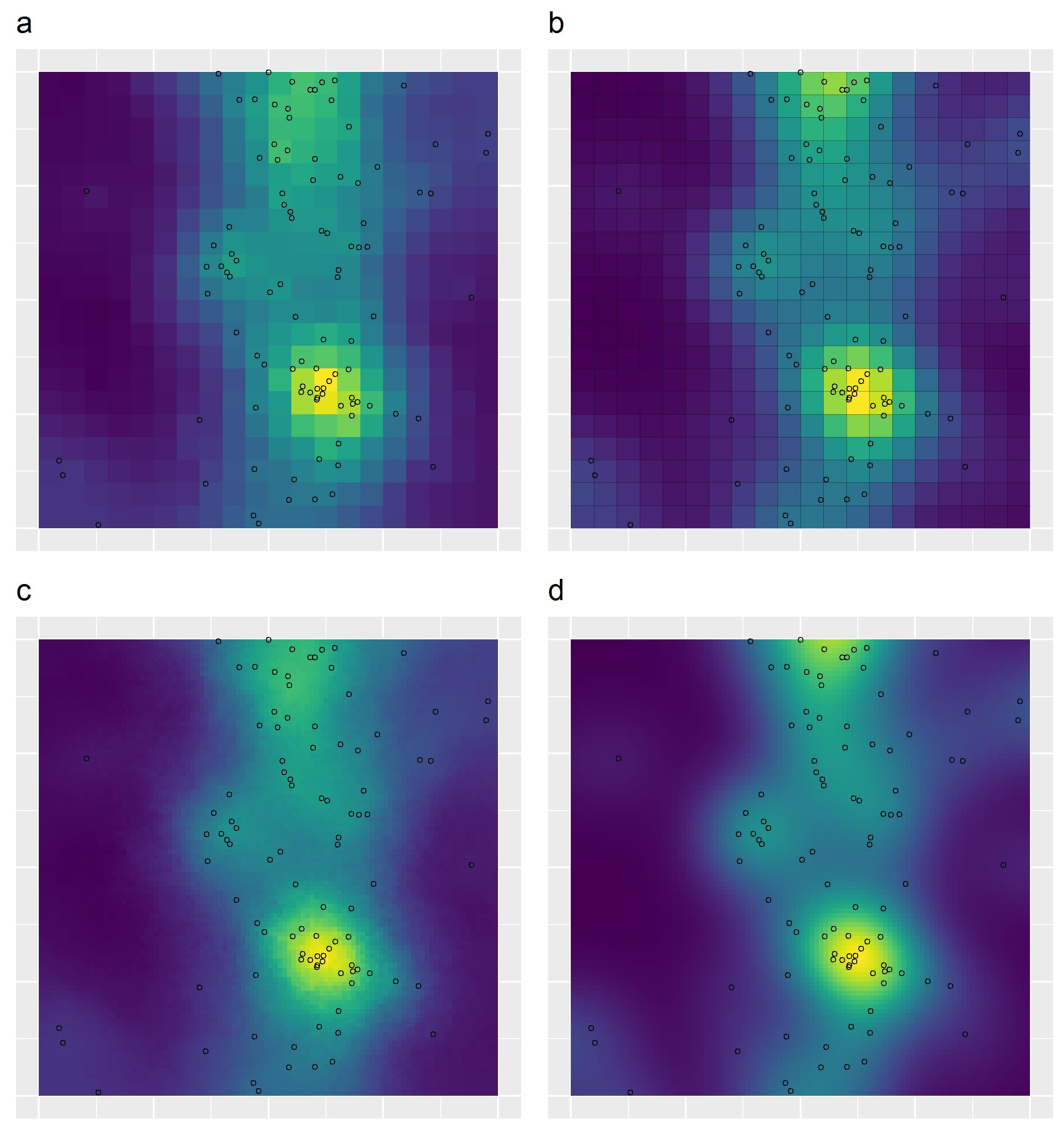


**Supplementary Fig. S7.** Targeting and logistical efficiency curves for OAM and a kernel-smoothed technique (KS). **a** Targeting efficiency curves. **b** Logistical efficiency curves. The minimal-resolution and single-aggregation curves (Fig. 2; main text) are reproduced in both plots, for reference.


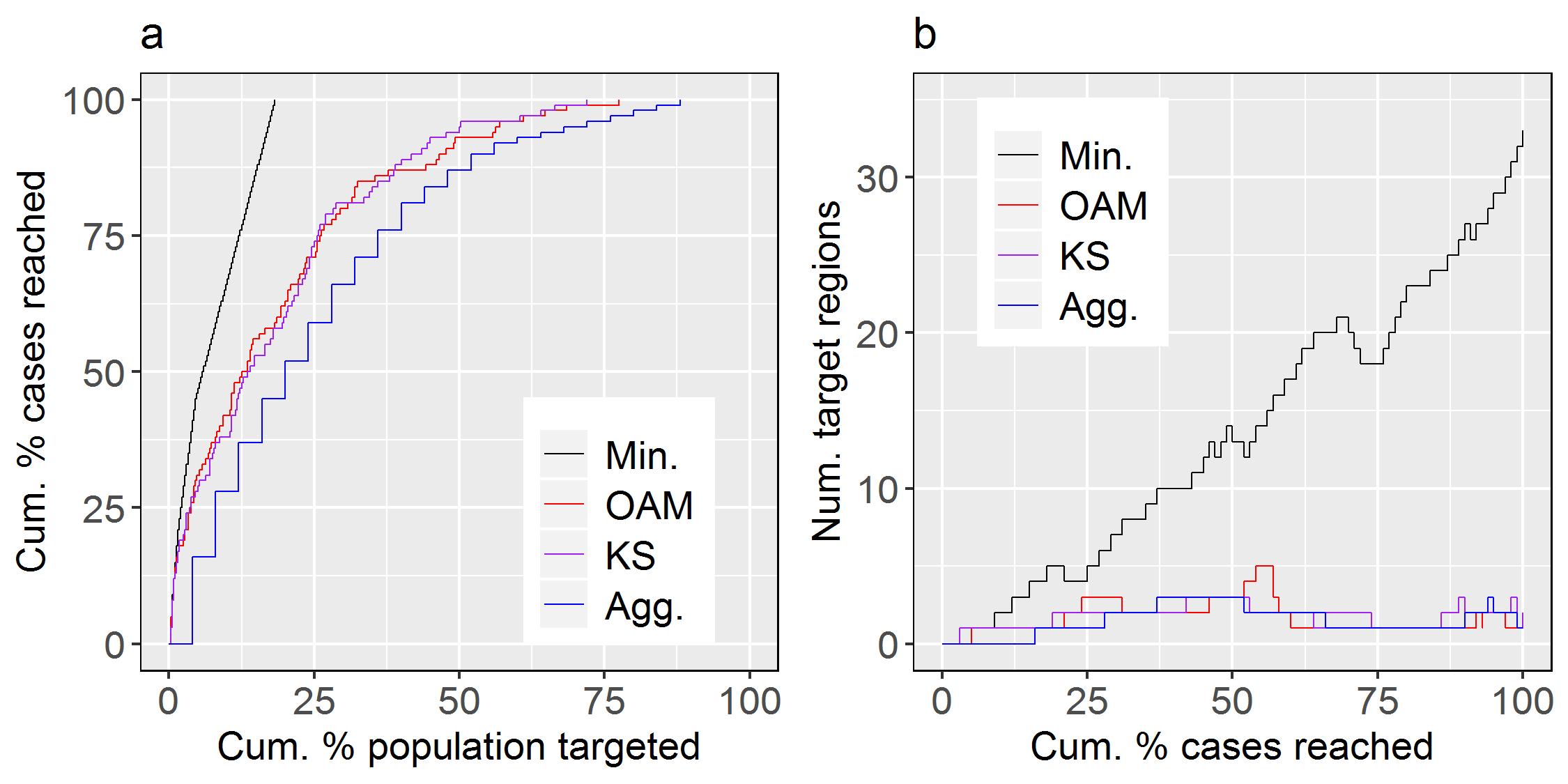


**Justification of the zonation-dependence confidence level**

A 64% confidence level was chosen in order to observe minimal unit hotspot counts ranging between zero and 100, i.e. the full dynamic range of possible values for that quantity. This allows the impact of the MAUP to be fully described. By contrast, using a higher limit, e.g. 95%, as is common practice, would have resulted in all minimal units having hotspot counts less than 100. This situation represents an exacerbation of the ZDN problem described in Results section ‘Global and local zonation dependence’ of the main text. Since the choice of confidence level affects only the investigation of the MAUP’s impact, and has no bearing on the map ultimately produced by OAM or its associated efficiency, it is left to the user. However, as a rule of thumb, we suggest a confidence level be chosen such that the full dynamic range of the minimal unit hotspot counts is observed, as we have done.

**Crude rate analysis of stroke**

As for the simulated dataset, the SA1 and SA2 values derived in this analysis are crude rates, while the minimal-resolution value derived using OAM is a population-weighted mean crude rate. The aggregate-level zonations used within OAM are the same as those used in the BYM model analysis (see main text).

Supplementary Fig. S8 shows the map produced using OAM. Supplementary Figs. S9a-b show the targeting and logistical efficiency curves, respectively, for the SA1, SA2, and OAM strategies. Note: the corresponding BYM analysis curves (Fig. 2; main text) are also shown, for reference. These curves demonstrate reduced targeting efficiency but increased logistical efficiency for the crude rate analysis, compared to the BYM model results. This is due to the additional smoothing of the underlying data that occurs when fitting the BYM model with OAM.

Supplementary Fig. S10 shows logistical efficiency maps for the three strategies based on the target case percentage of 15%, and Supplementary Table S1 the corresponding exact efficiency data.

**Supplementary Fig. S8.** Map of population-weighted mean crude rates of stroke produced using OAM.


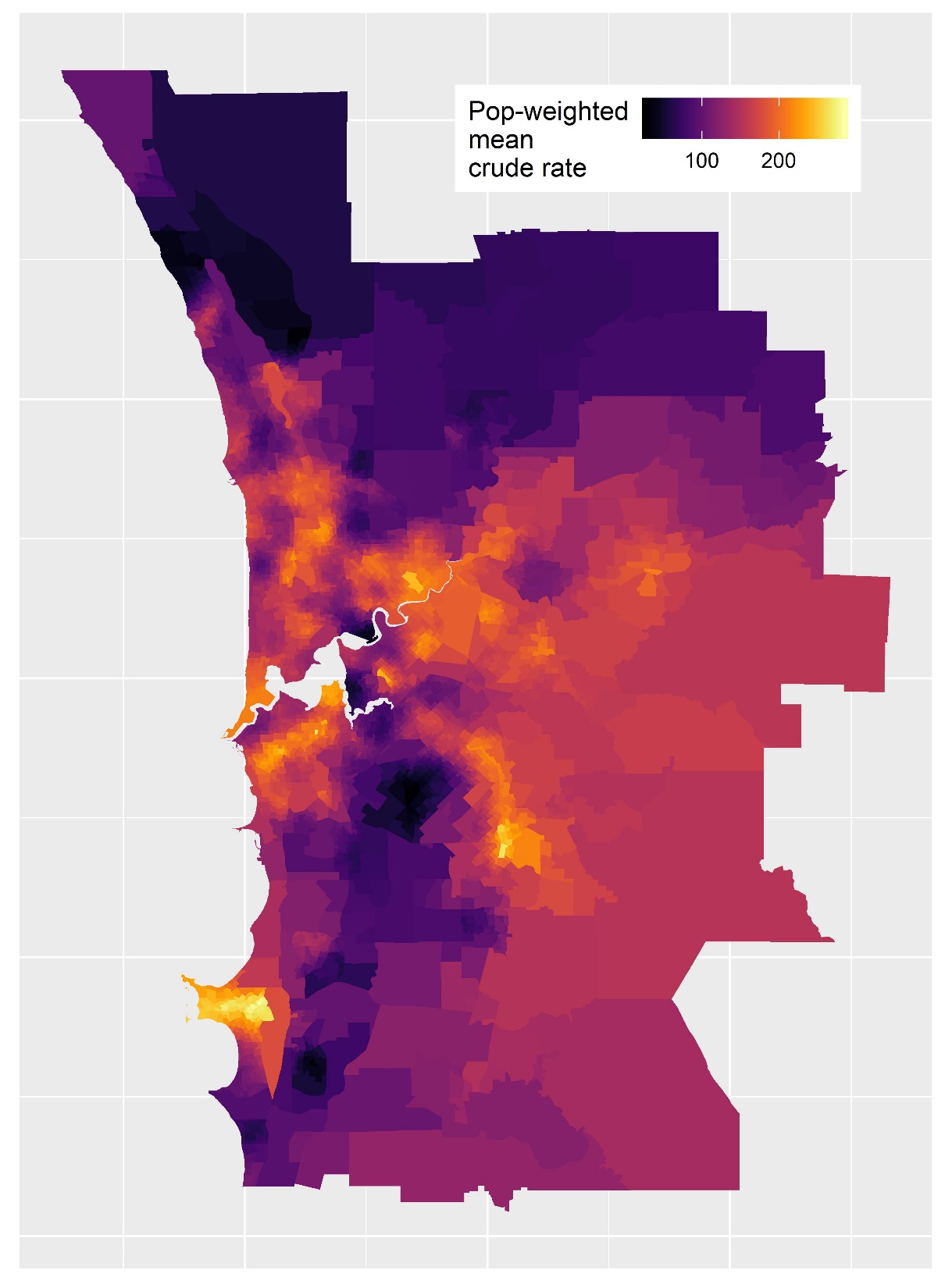


**Supplementary Fig. S9.** Targeting and logistical efficiency curves for stroke. **a** Targeting efficiency curves. **b** Logistical efficiency curves.


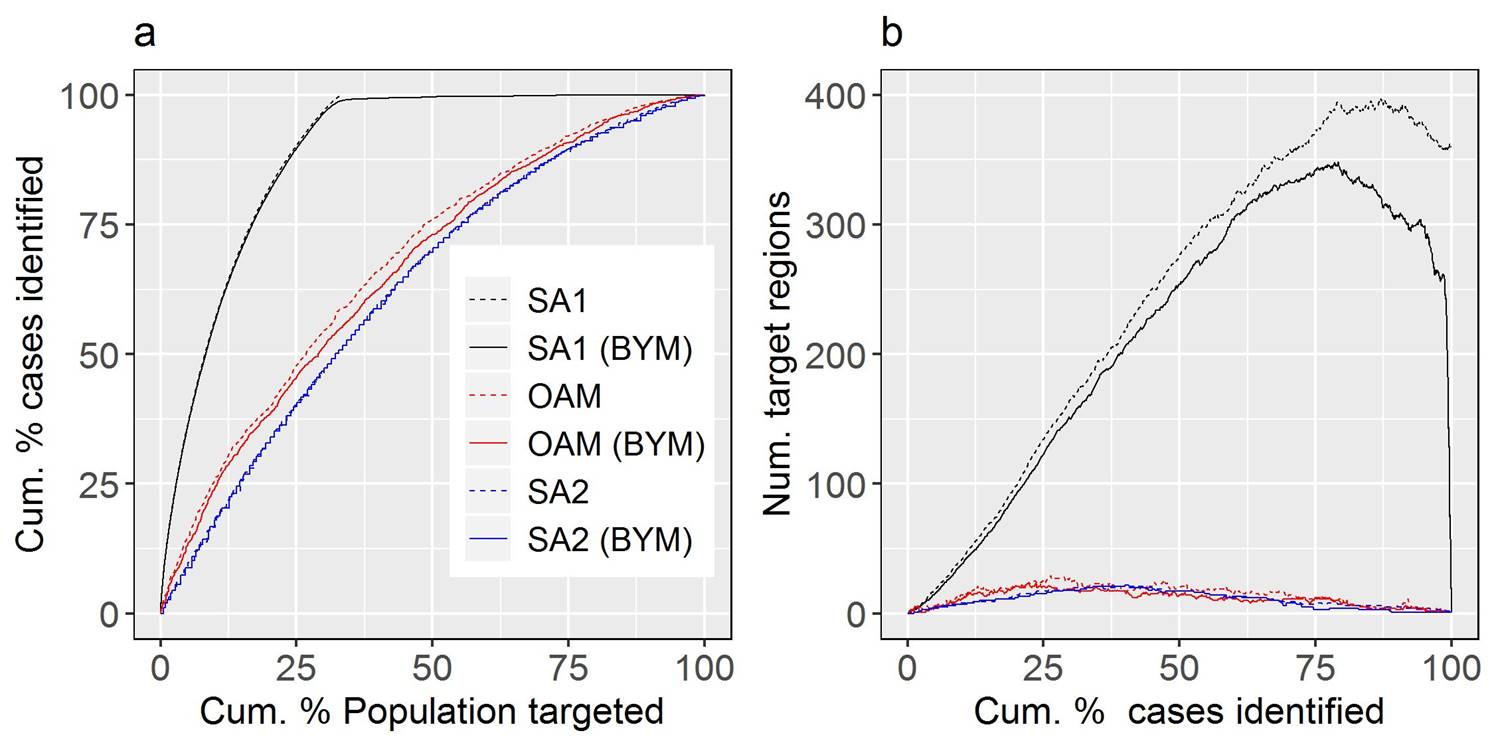


**Supplementary Fig. S10.** Logistical efficiency maps for the crude rate analysis of stroke based on the target case percentage of 15%. **a** SA1 map. **b** SA2 map. **c** OAM map.


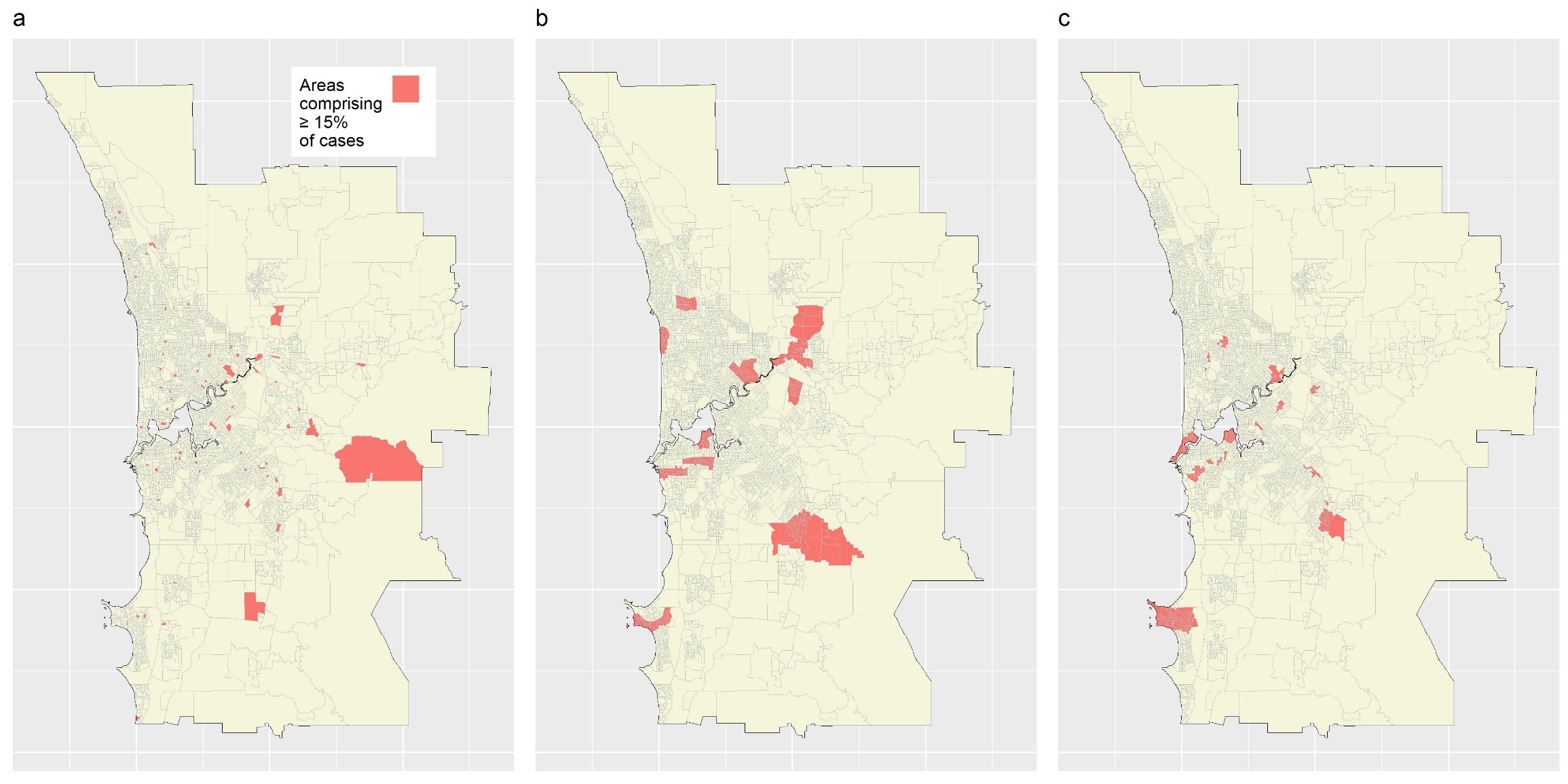


**Supplementary Table S1.** Exact efficiency data for the crude rate analysis of stroke, based on the target case percentage of 15%.

| **Mapping method** | **Cum. % of population targeted** | **Number of target regions** |
| --- | --- | --- |
| **SA1** | 1.4 | 70 |
| **OAM** | 5.3 | 18 |
| **SA2** | 8.6 | 10 |

**Stabilisation of population sizes**

Differences in population sizes among units is one of several criteria used in evaluating the appropriateness of geographical units used in ecological studies, with similarity in population sizes being preferred [S1]. Supplementary Fig. S11 shows the 2016 population size distributions of SA1, SA2, and units within OAM’s zonations for the stroke analysis. The SA1 population sizes ranged between zero and 3,603 and were generally tightly distributed around a mean of approximately 435. By contrast, population sizes among SA2s varied widely between zero and 36,128, with a mean of 11,256. This illustrates a common issue present but seldom acknowledged in practice: by aggregating fine-resolution data by pre-defined, coarse-resolution administrative units, one does not always escape small-number issues. OAM overcomes this, with population sizes among units in its zonations ranging between 10,037 and 12,984 and having a mean of 11,342.

**Supplementary Fig. S11.** 2016 population size distributions for different spatial units in Perth. **a** SA1. **b** SA2. **c** Units within OAM’s zonations.


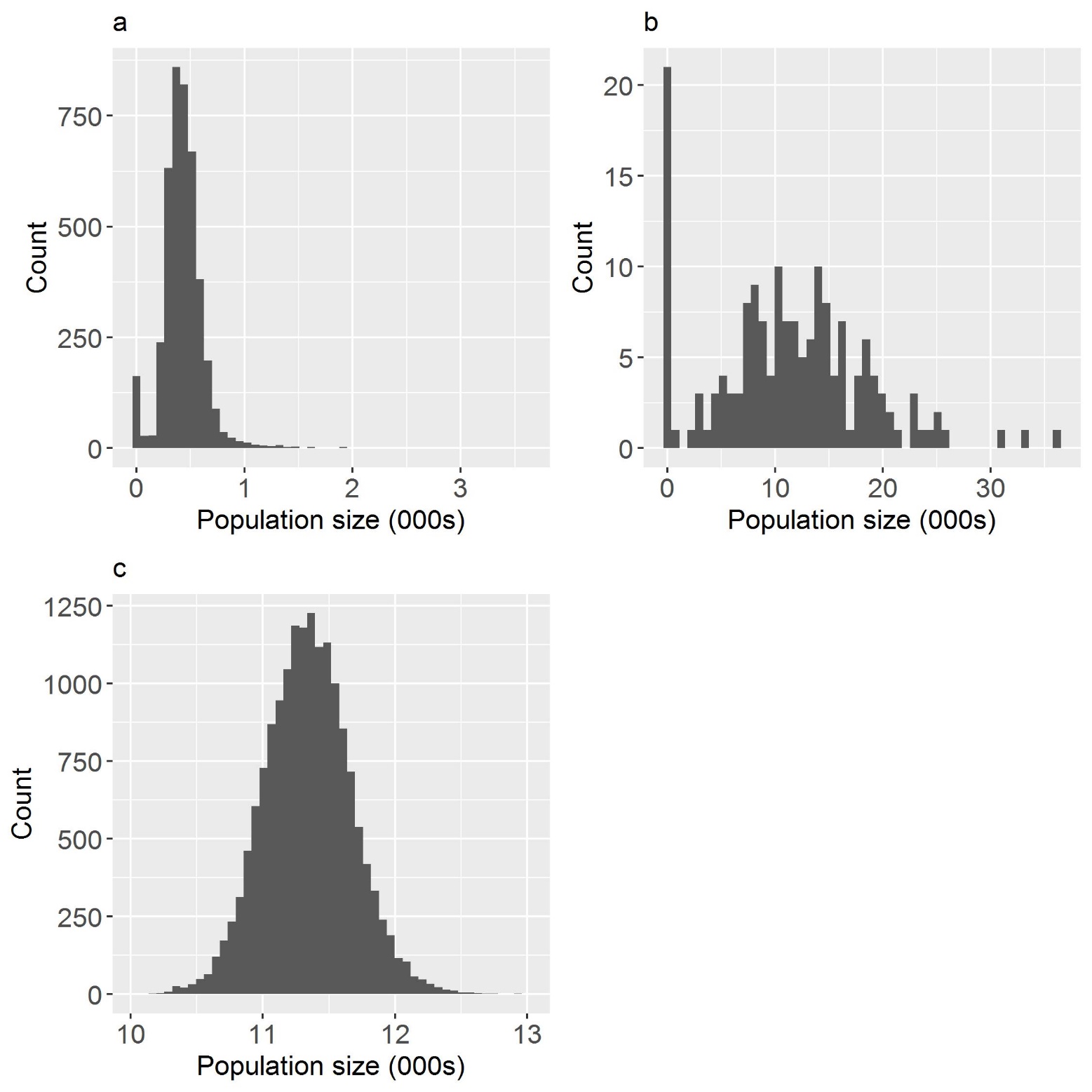


**Supplementary references**

S1. Arsenault J, Michel P, Berke O, Ravel A, Gosselin P. How to choose geographical units in ecological studies: Proposal and application to campylobacteriosis. Spatial and spatio-temporal epidemiology. 2013; 7:11-24.
